# Supplementary material for: Cord Placement Model: An Instructional Guide for Preclinical Dental Students to Practice the Skill of Retraction Cord Placement
Source: MedEdPORTAL. 2023 Feb 28;19:11303. doi: 10.15766/mep_2374-8265.11303 (PMC9971216; doi:10.15766/mep_2374-8265.11303)
Supplement: Supplementary file 1 — Retraction Cord Model Instructional Guide.mp4Instructional Guide for Model Fabrication.docxStudents Instructional Guide.docxFaculty Survey.docxGingival Displacement With Retraction Cord.pptxStudents Instructional Guide Video.mp4Implementation Guide.docxCord Packing Assessment.docxD3 Student Survey.docxD4 Student Survey.docx [file mep_2374-8265.11303-s001.zip › B. Instructional Guide for Model Fabrication.docx]

**Step-by-step Instructional Guide for Fabricating a Retraction Cord Packing Model**


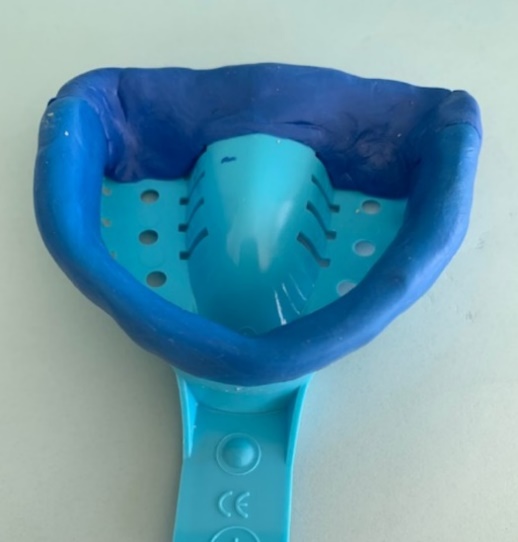


**Step 1.** Extend border of stock tray with PVS putty.


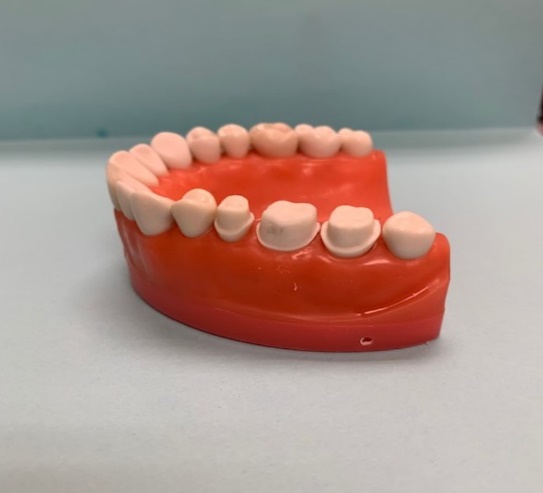


**Step 2.** Prepare selected typodont teeth for crowns.


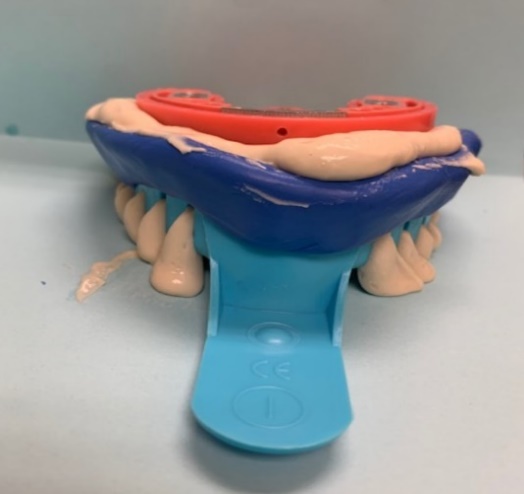


**Step 3.** Take alginate impression of the typodont in prepared tray.


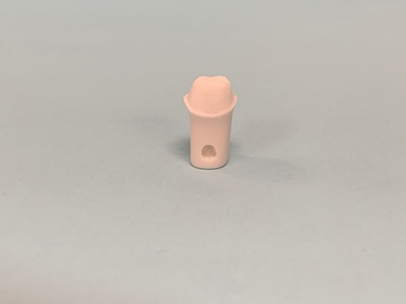


**Step 4.** Before pouring up the stone model, it is best to create a retention point in the root portion of the typodont tooth.


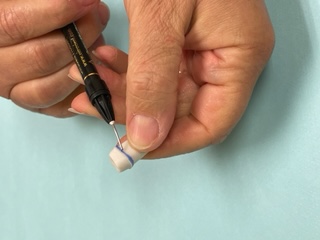


**Step 5.** Apply lubricant to prevent block out resin from sticking to the plastic tooth. Apply block out resin 1mm below margin of the prep to create a sulcus.


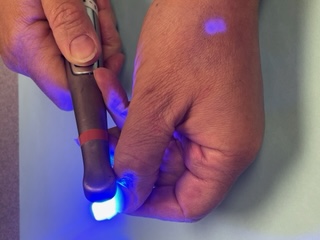


**Step 6.** Cure block out resin.


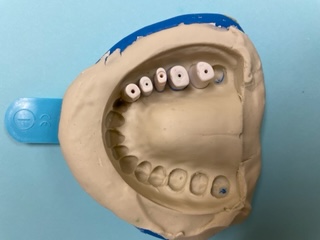


**Step 7.** Insert teeth back into the alginate impression.


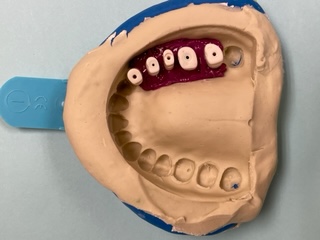


**Step 8.** Inject heavy or medium body PVS impression material around prepared teeth with small tip.


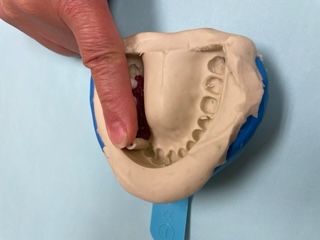


**Step 9.** Remove section, inspect and refill imperfect areas with PVS before repositioning section back into the alginate impression with finger pressure until set.


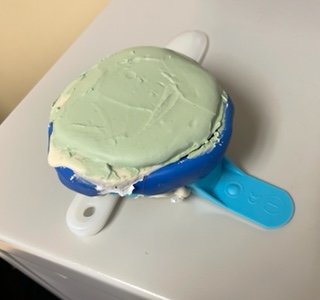


**Step 10.** Pour up with jade stone.


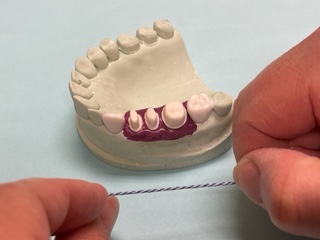


**Step 11.** Model is now ready for retraction cord packing.

Photography by Dr. Chu Fountain
