# Supplementary material for: Cord Placement Model: An Instructional Guide for Preclinical Dental Students to Practice the Skill of Retraction Cord Placement
Source: MedEdPORTAL. 2023 Feb 28;19:11303. doi: 10.15766/mep_2374-8265.11303 (PMC9971216; doi:10.15766/mep_2374-8265.11303)
Supplement: Supplementary file 1 — Retraction Cord Model Instructional Guide.mp4Instructional Guide for Model Fabrication.docxStudents Instructional Guide.docxFaculty Survey.docxGingival Displacement With Retraction Cord.pptxStudents Instructional Guide Video.mp4Implementation Guide.docxCord Packing Assessment.docxD3 Student Survey.docxD4 Student Survey.docx [file mep_2374-8265.11303-s001.zip › C. Students Instructional Guide.docx]

**Students’ Instructional Guide for Packing Retraction Cord**


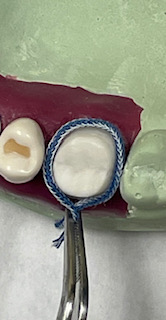


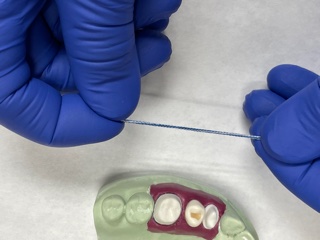


**Step 2.** Form a loop of retraction cord around the tooth and hold it taut with the thumb and forefinger or with a cotton plier.

**Step 4.** As the cord is placed subgingivally, the instrument must be pushed slightly towards the area already tucked into place. If the force of the instrument is directed away from the area previously packed, the cord already packed will be pulled out.

**Step 3.** Start the placement of the retraction cord by gently pushing it into the sulcus on the mesial surface of the tooth.

**Step 1.** Cut off a 2 inches piece of retraction cord, size 0 or 00 depending on which tooth.


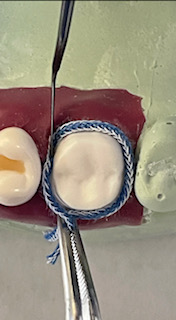

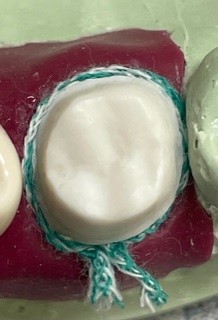


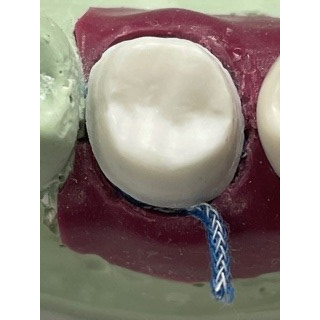


Photography by Dr. Chu Fountain

**Step 6.** When using a second larger cord, part of it is placed subgingivally and the top part should be visible supragingival.

**Step 5**. Cut off excess cord when two ends approximate each other (<1mm). Retraction cord should be subgingival at this point.

Shillingburg HT, Sather DA, Wilson EL, et al. *Fundamentals of Fixed Prosthodontics.*4^th^ ed.   Hanover Park, IL: Quintessence Publishing Co, Inc
