# Supplementary material for: Cord Placement Model: An Instructional Guide for Preclinical Dental Students to Practice the Skill of Retraction Cord Placement
Source: MedEdPORTAL. 2023 Feb 28;19:11303. doi: 10.15766/mep_2374-8265.11303 (PMC9971216; doi:10.15766/mep_2374-8265.11303)
Supplement: Supplementary file 1 — Retraction Cord Model Instructional Guide.mp4Instructional Guide for Model Fabrication.docxStudents Instructional Guide.docxFaculty Survey.docxGingival Displacement With Retraction Cord.pptxStudents Instructional Guide Video.mp4Implementation Guide.docxCord Packing Assessment.docxD3 Student Survey.docxD4 Student Survey.docx [file mep_2374-8265.11303-s001.zip › D. Faculty Survey.docx]

**Faculty Survey**

**Faculty perception in the assessment of the retraction cord model as an instructional tool survey**

Please respond to the following questions based on the Likert scale of 1-5.

1 – strongly agree

2 – agree

3 – no opinion

4 – disagree

5 – strongly disagree

1. The model is representative of the experience of placing retraction cord in a patient.

2. The written instructional guide is beneficial adjunct to the model.

Please use the 1 to 4 rating scale for the next two questions.

1 - Excellent

2 - Good

3 - Fair

4 - Poor

3. What is your overall rating of the model and instructional guide?

4. How would you rate the student’s overall experience of using the instructional model and guide?

5. What suggestion do you have to improve the instructional model and guide?

_________________________________________________________________________________
