# Supplementary material for: Cord Placement Model: An Instructional Guide for Preclinical Dental Students to Practice the Skill of Retraction Cord Placement
Source: MedEdPORTAL. 2023 Feb 28;19:11303. doi: 10.15766/mep_2374-8265.11303 (PMC9971216; doi:10.15766/mep_2374-8265.11303)
Supplement: Supplementary file 1 — Retraction Cord Model Instructional Guide.mp4Instructional Guide for Model Fabrication.docxStudents Instructional Guide.docxFaculty Survey.docxGingival Displacement With Retraction Cord.pptxStudents Instructional Guide Video.mp4Implementation Guide.docxCord Packing Assessment.docxD3 Student Survey.docxD4 Student Survey.docx [file mep_2374-8265.11303-s001.zip › E. Gingival Displacement With Retraction Cord.pptx]

## Slide 1
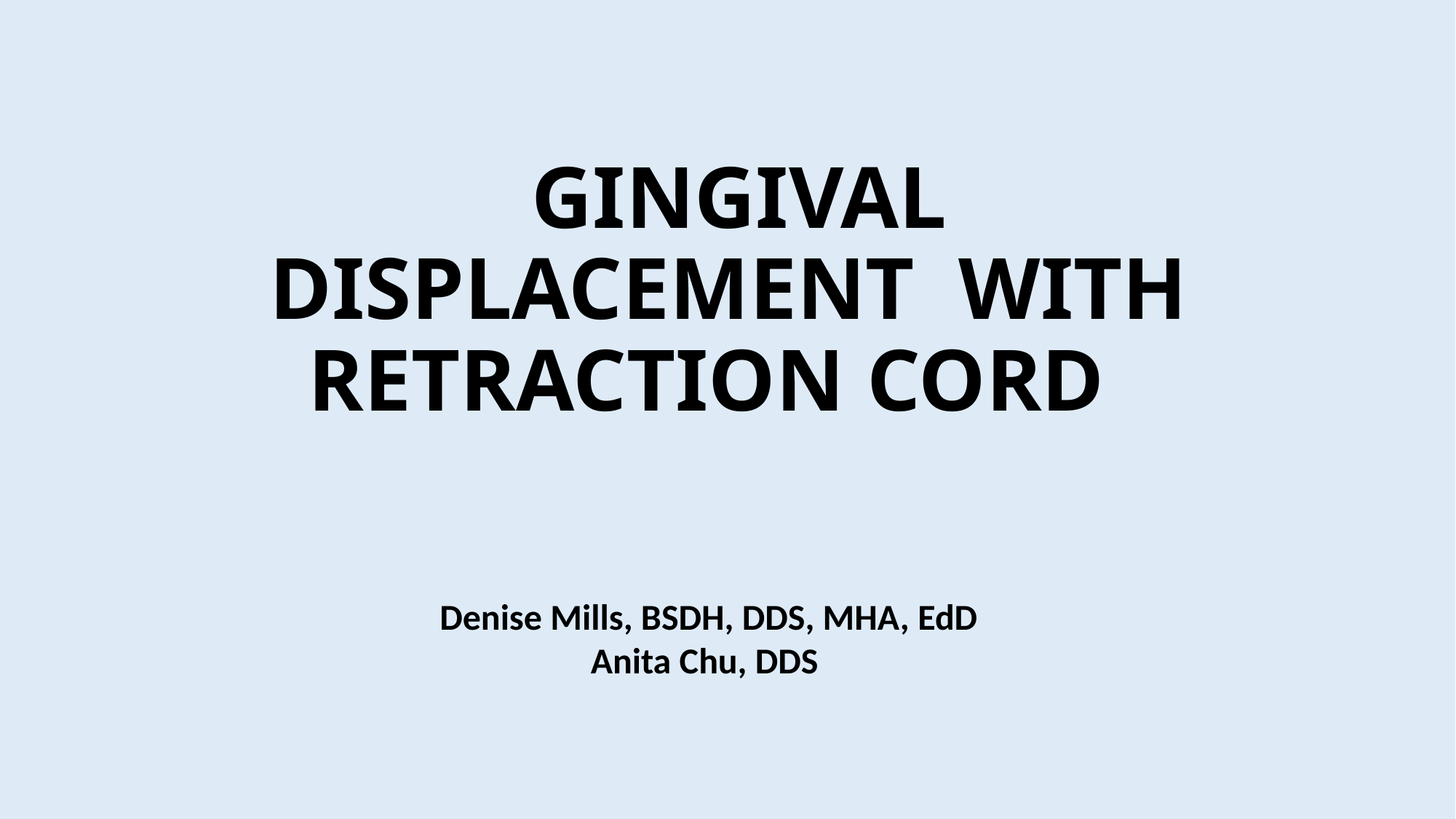

# GINGIVAL DISPLACEMENT  WITH RETRACTION CORD
Denise Mills, BSDH, DDS, MHA, EdD
Anita Chu, DDS

## Slide 2
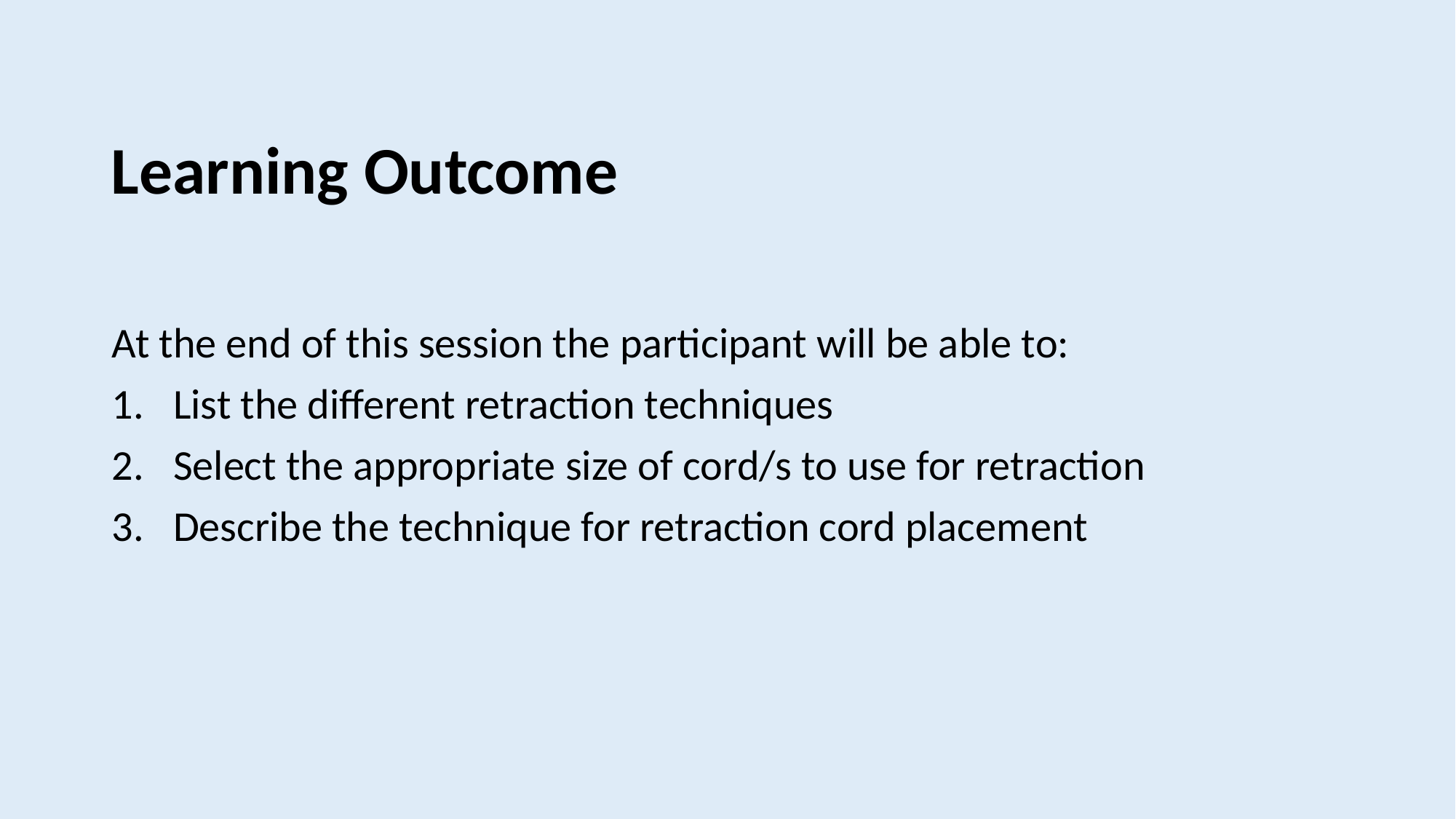

# Learning Outcome
At the end of this session the participant will be able to:
List the different retraction techniques
Select the appropriate size of cord/s to use for retraction
Describe the technique for retraction cord placement

## Slide 3
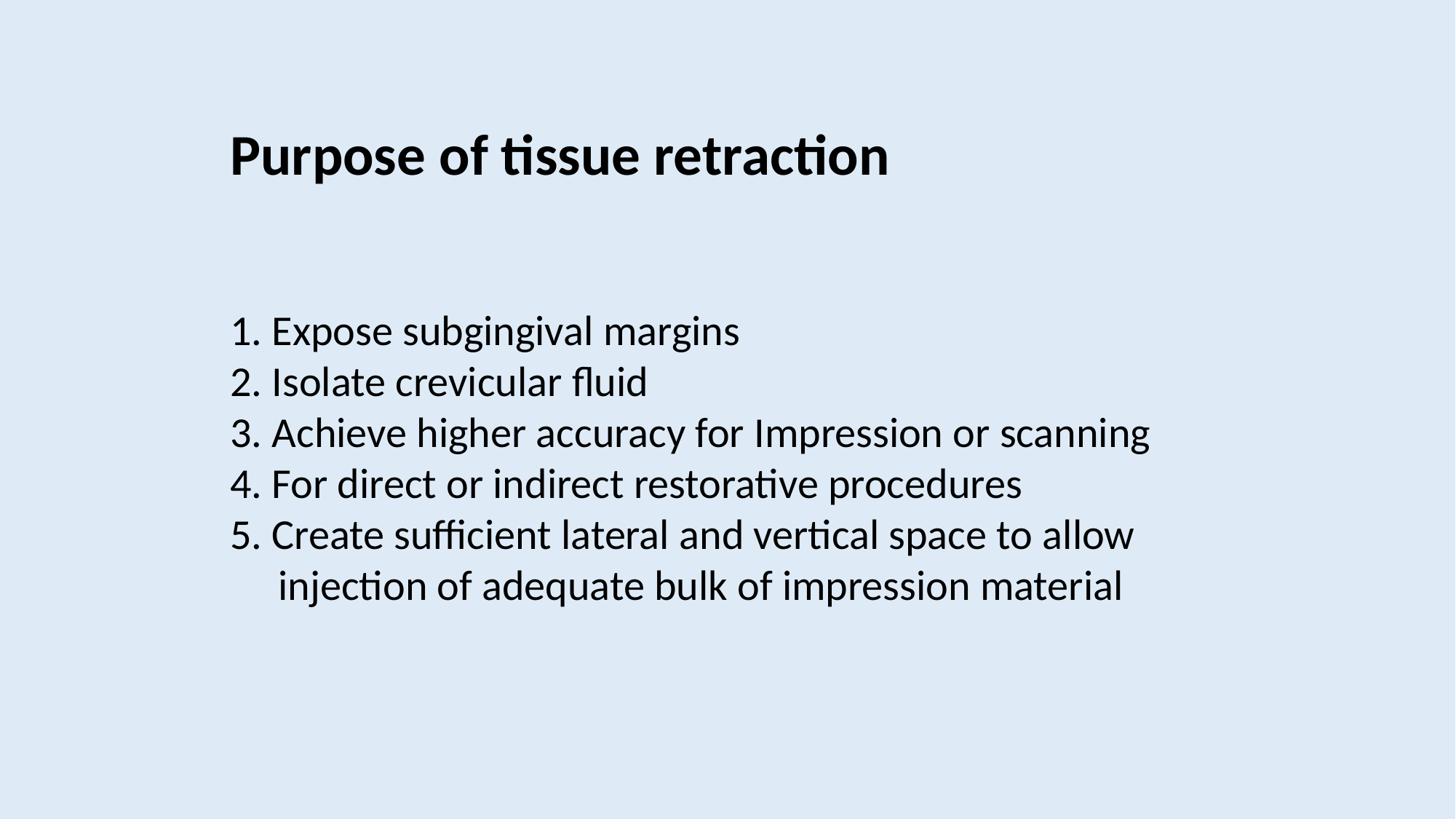

Purpose of tissue retraction
1. Expose subgingival margins
2. Isolate crevicular fluid
3. Achieve higher accuracy for Impression or scanning
4. For direct or indirect restorative procedures
5. Create sufficient lateral and vertical space to allow
     injection of adequate bulk of impression material

## Slide 4
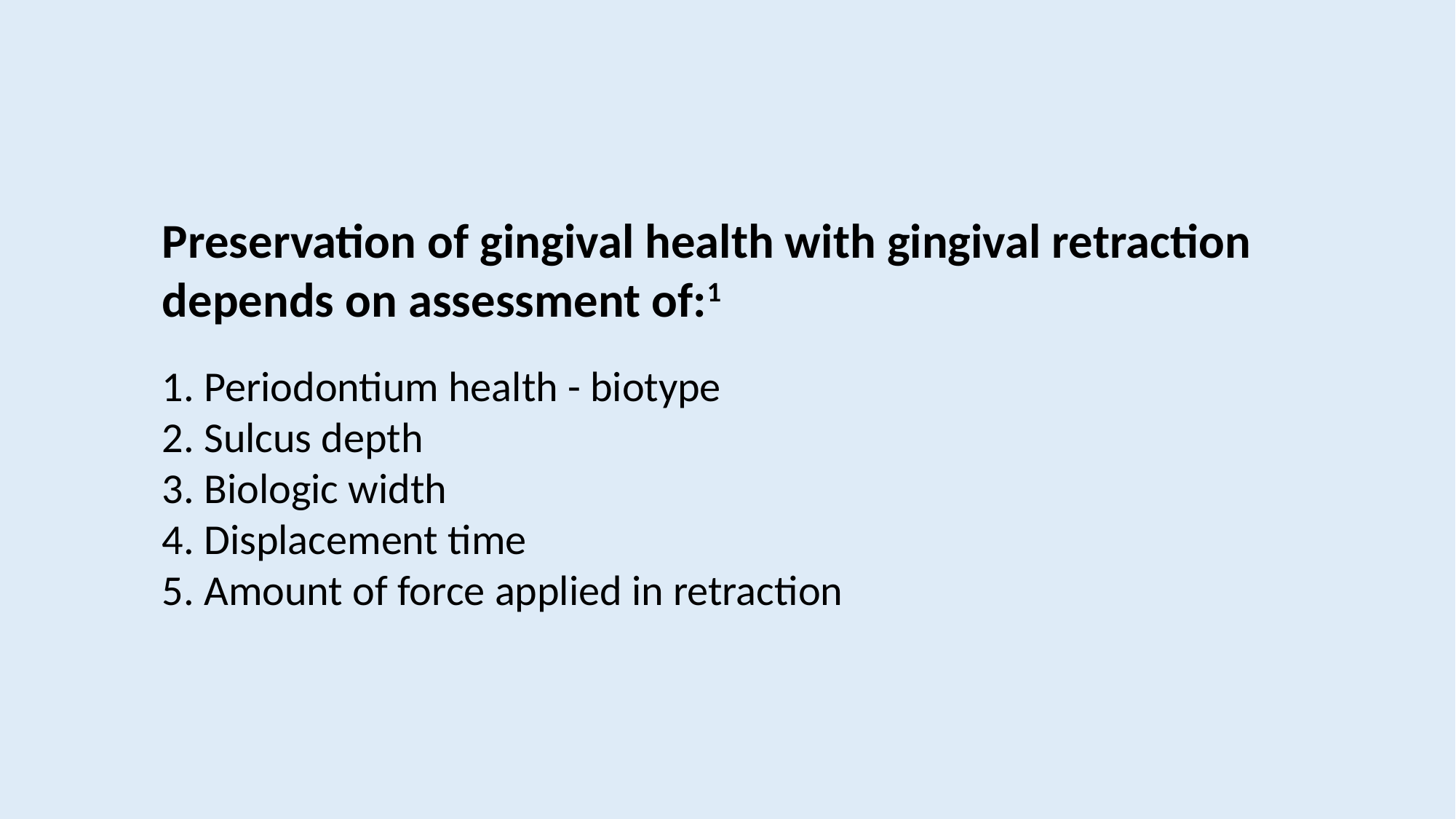

Preservation of gingival health with gingival retraction depends on assessment of:1
1. Periodontium health - biotype
2. Sulcus depth
3. Biologic width
4. Displacement time
5. Amount of force applied in retraction

## Slide 5
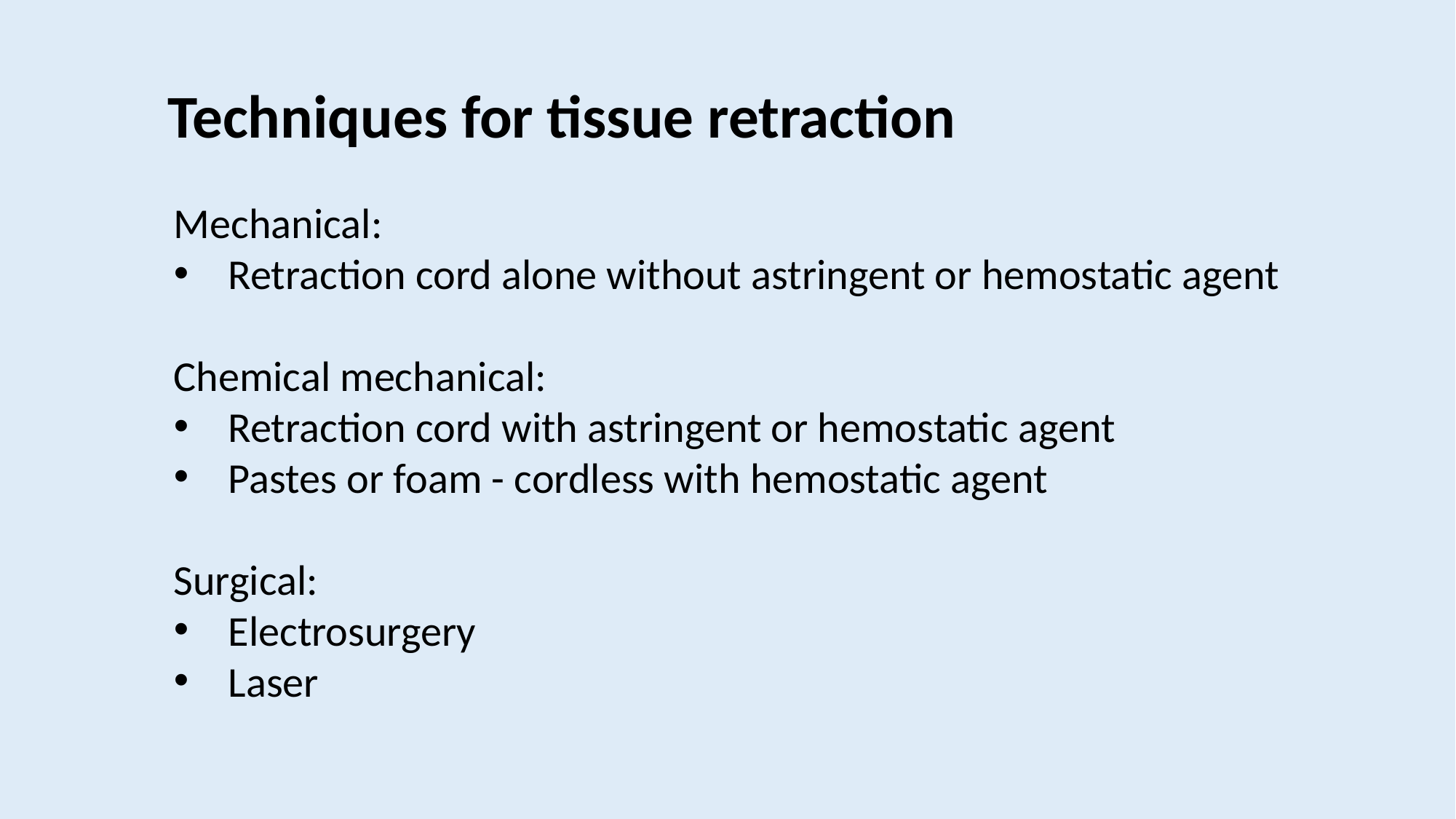

Techniques for tissue retraction
Mechanical:
Retraction cord alone without astringent or hemostatic agent
Chemical mechanical:
Retraction cord with astringent or hemostatic agent
Pastes or foam - cordless with hemostatic agent
Surgical:
Electrosurgery
Laser

## Slide 6
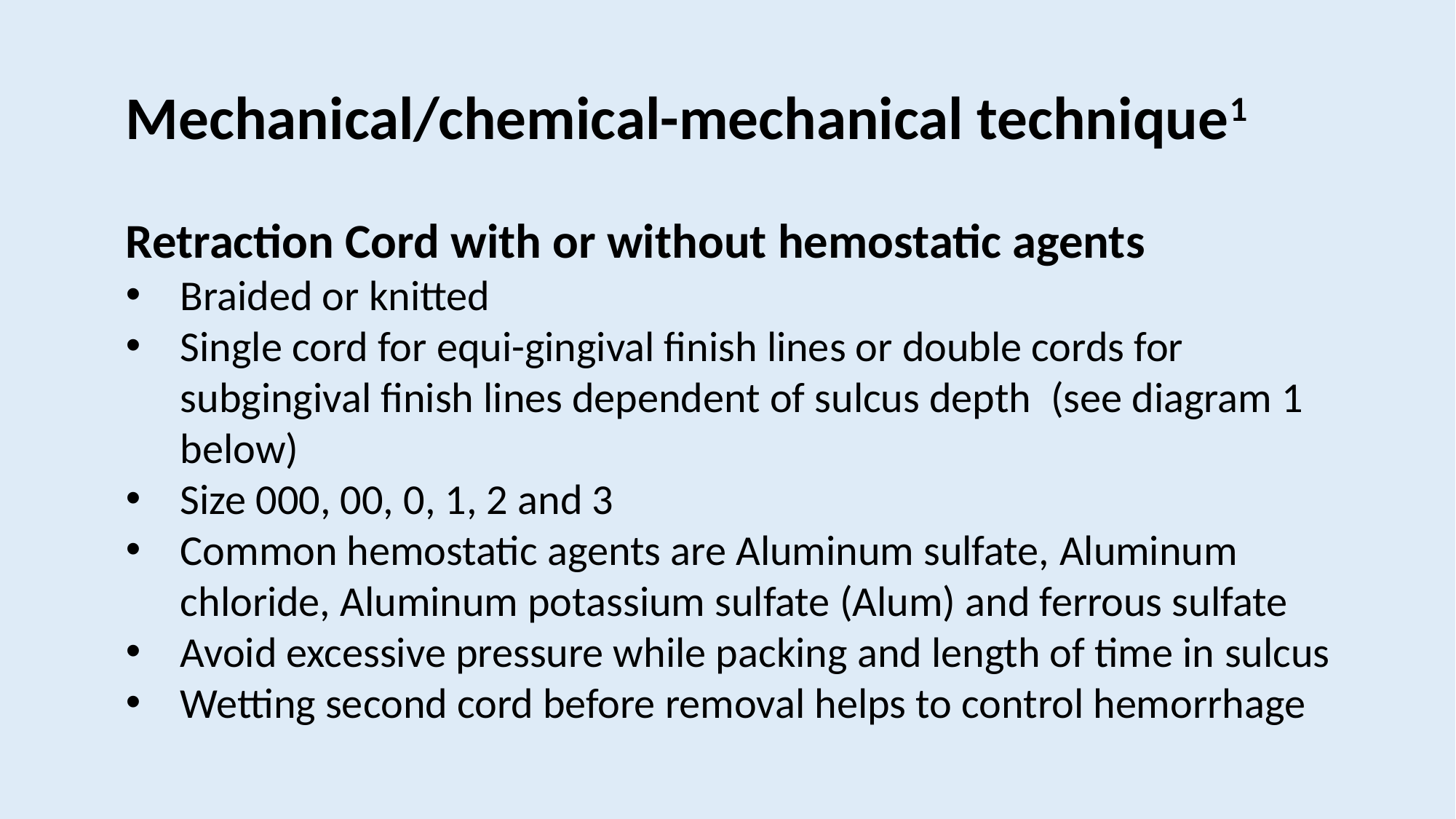

Mechanical/chemical-mechanical technique1
Retraction Cord with or without hemostatic agents
Braided or knitted
Single cord for equi-gingival finish lines or double cords for subgingival finish lines dependent of sulcus depth  (see diagram 1 below)
Size 000, 00, 0, 1, 2 and 3
Common hemostatic agents are Aluminum sulfate, Aluminum chloride, Aluminum potassium sulfate (Alum) and ferrous sulfate
Avoid excessive pressure while packing and length of time in sulcus
Wetting second cord before removal helps to control hemorrhage

## Slide 7
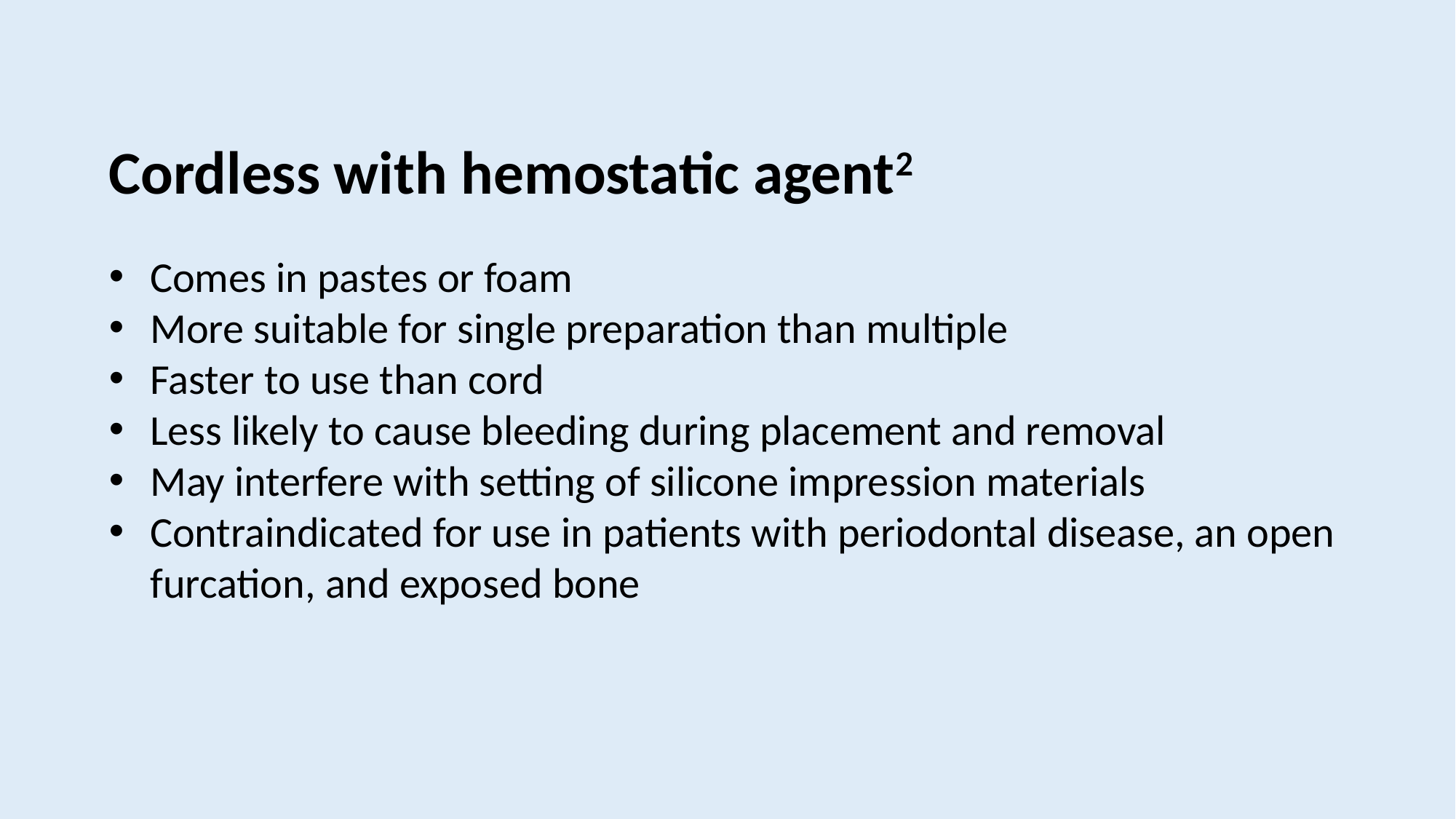

Cordless with hemostatic agent2
Comes in pastes or foam
More suitable for single preparation than multiple
Faster to use than cord
Less likely to cause bleeding during placement and removal
May interfere with setting of silicone impression materials
Contraindicated for use in patients with periodontal disease, an open furcation, and exposed bone

## Slide 8
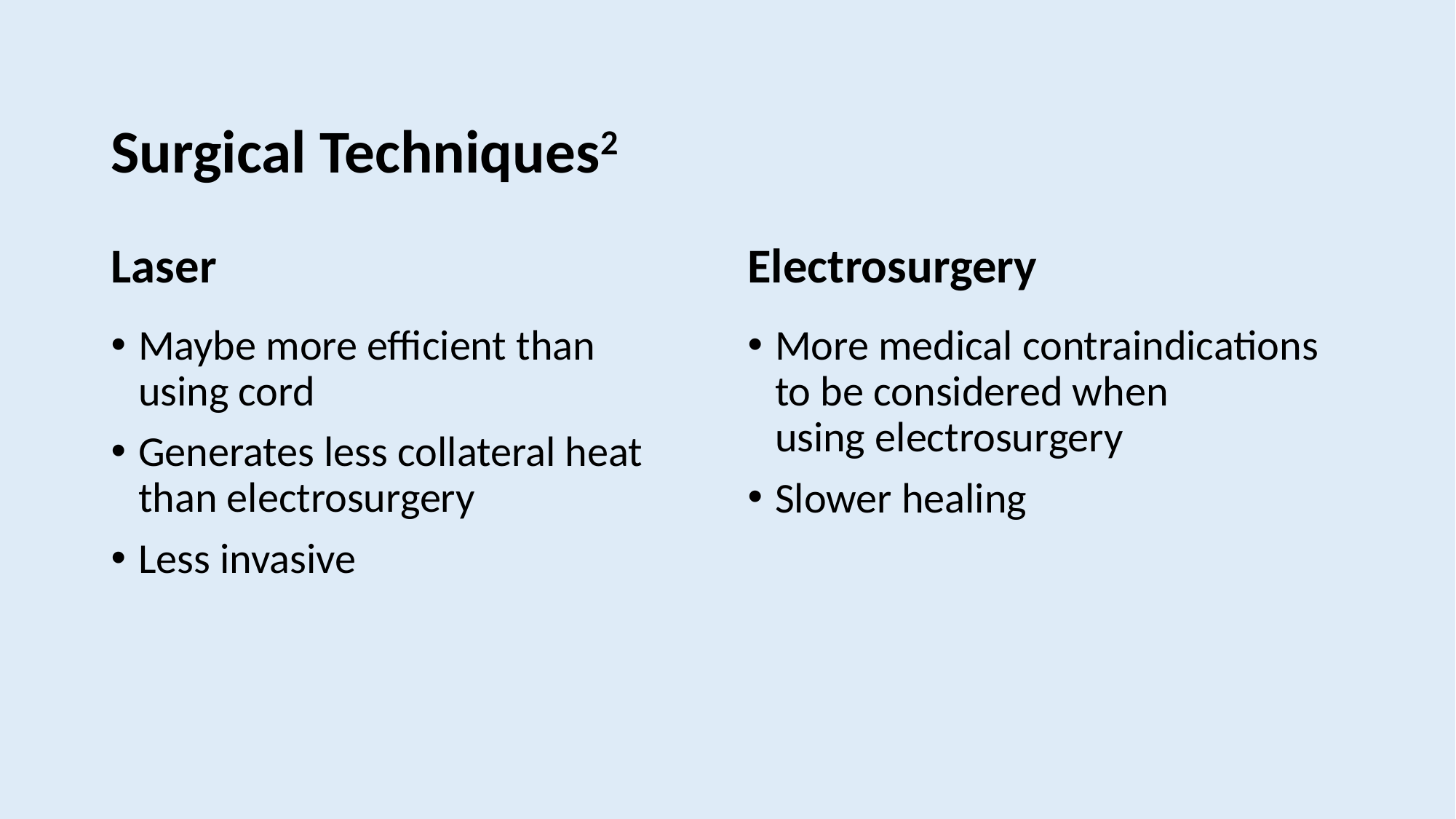

# Surgical Techniques2
Laser
Electrosurgery
Maybe more efficient than using cord
Generates less collateral heat than electrosurgery
Less invasive
More medical contraindications to be considered when using electrosurgery
Slower healing

## Slide 9
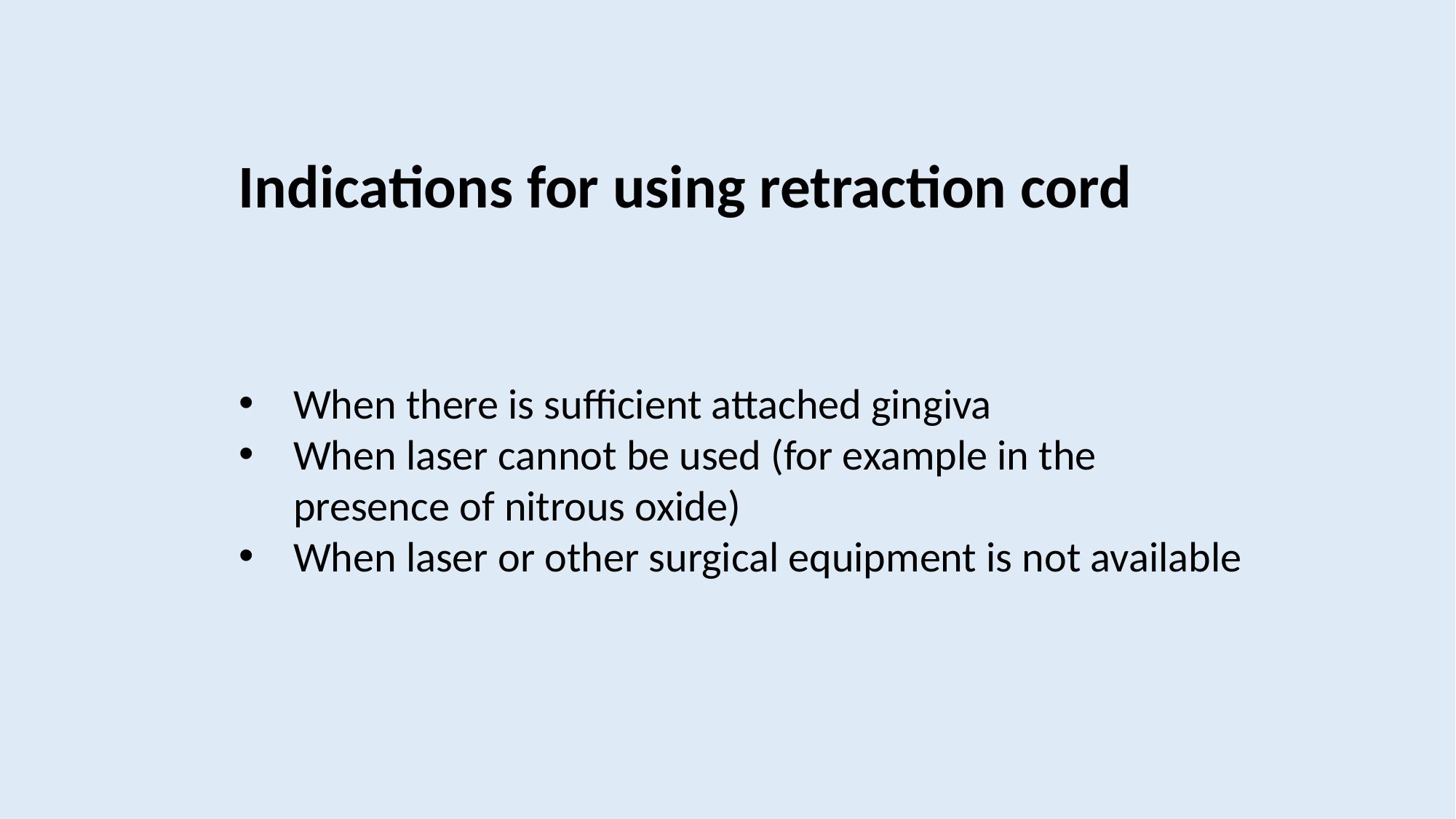

Indications for using retraction cord
When there is sufficient attached gingiva
When laser cannot be used (for example in the presence of nitrous oxide)
When laser or other surgical equipment is not available

## Slide 10
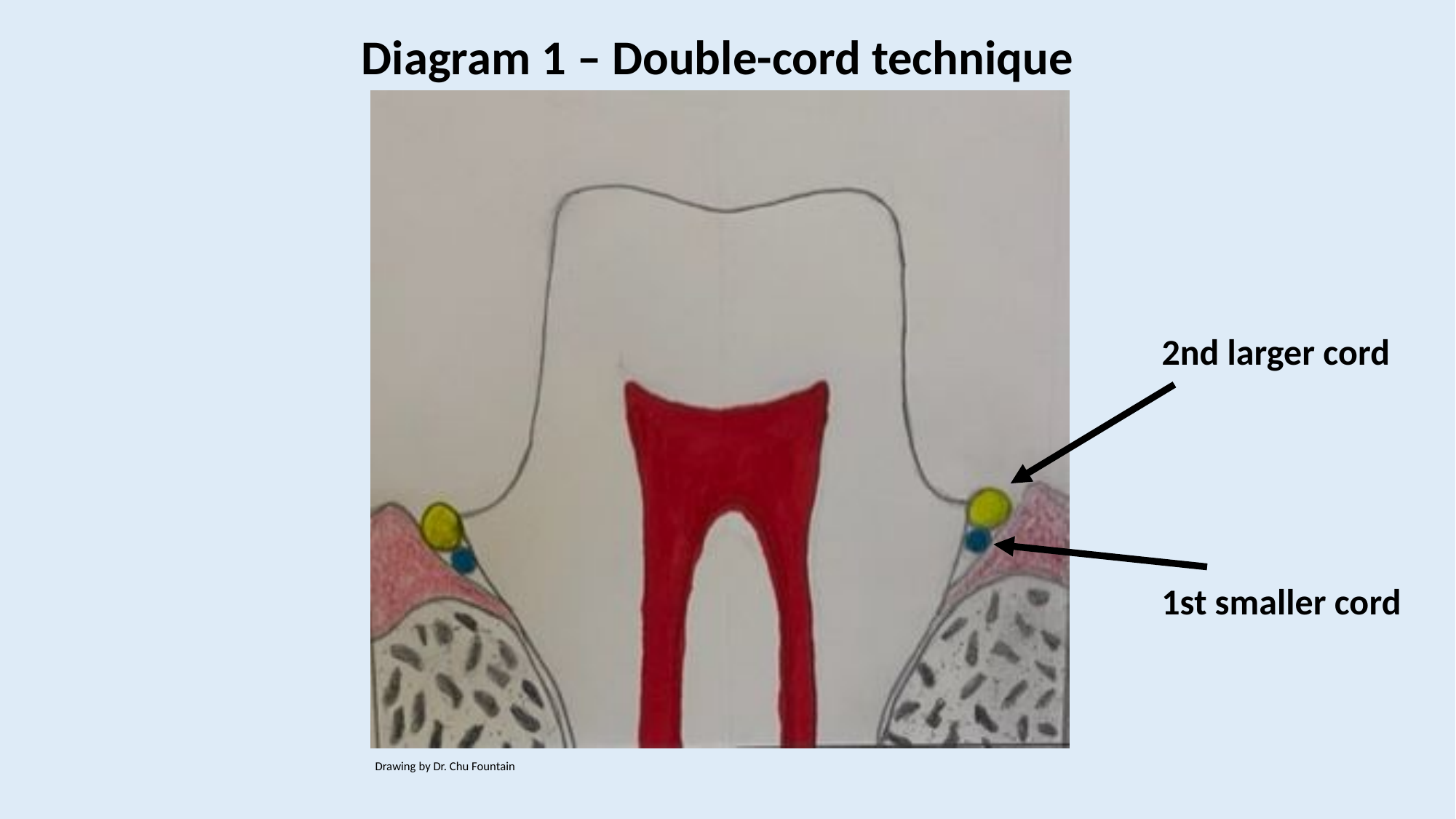

Diagram 1 – Double-cord technique
2nd larger cord
1st smaller cord
Drawing by Dr. Chu Fountain

## Slide 11
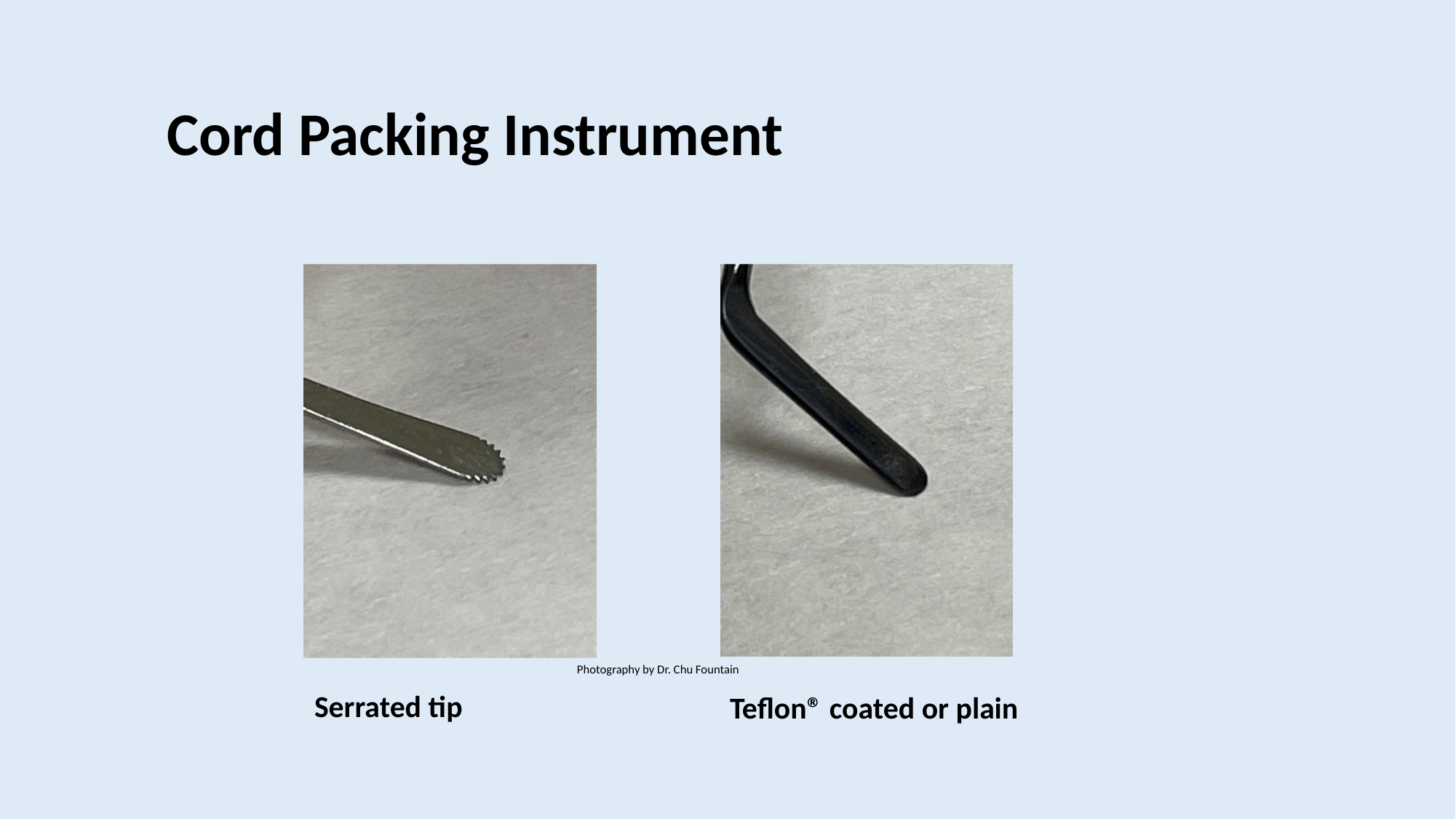

Cord Packing Instrument
Serrated tip
Photography by Dr. Chu Fountain
Teflon® coated or plain

## Slide 12
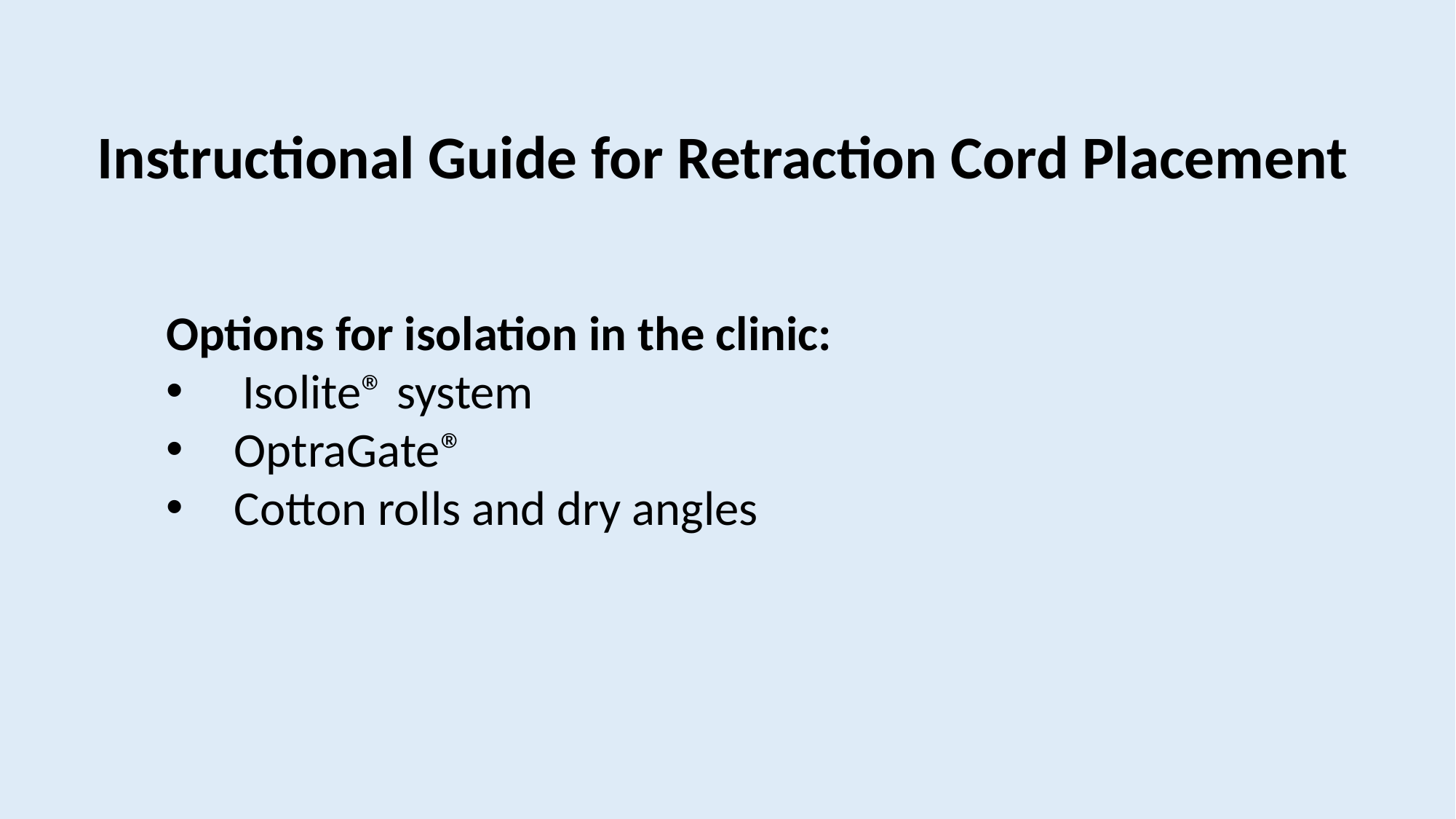

Instructional Guide for Retraction Cord Placement
Options for isolation in the clinic:
  Isolite® system
OptraGate®
Cotton rolls and dry angles

## Slide 13
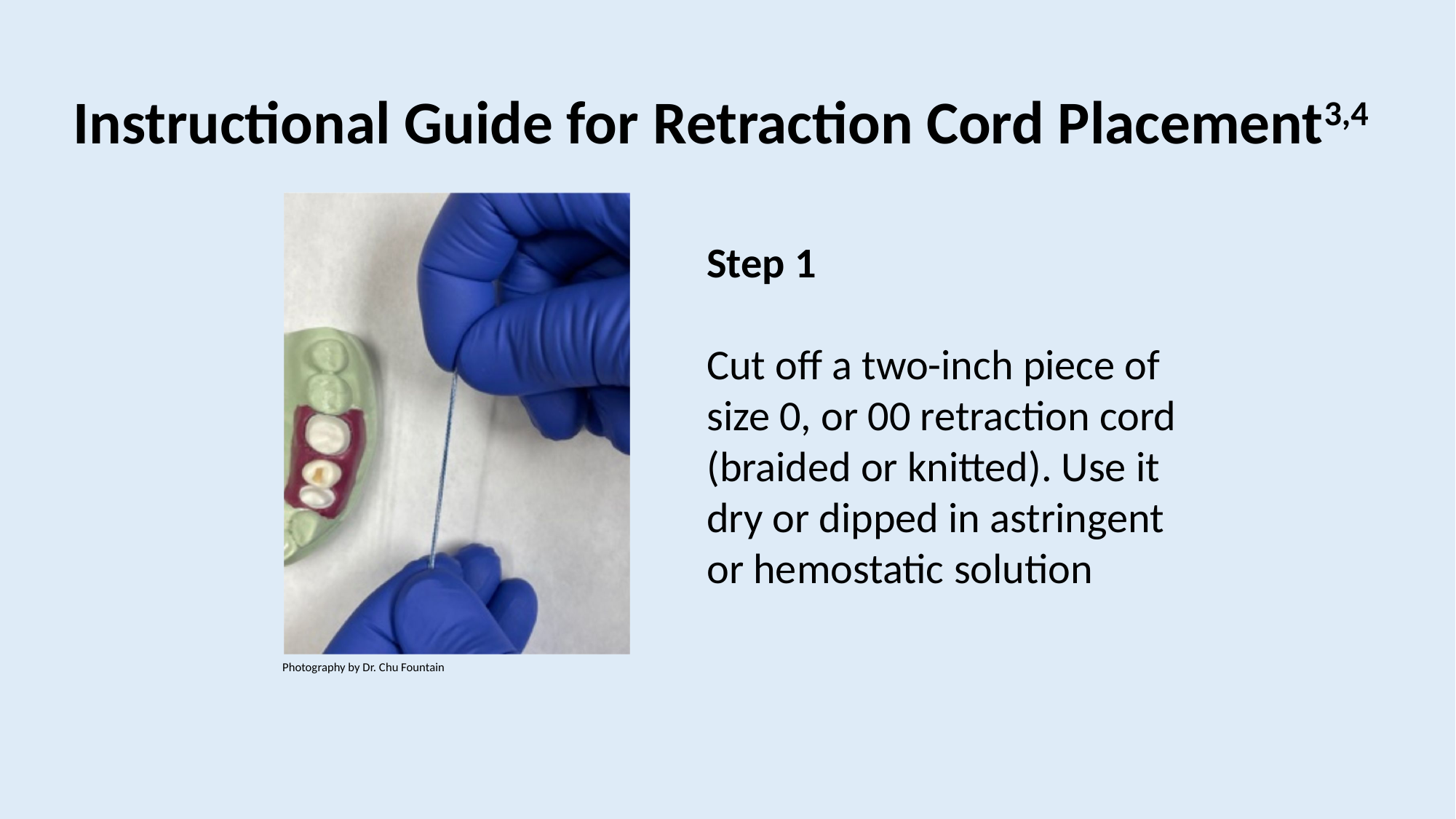

Instructional Guide for Retraction Cord Placement3,4
Step 1
Cut off a two-inch piece of size 0, or 00 retraction cord (braided or knitted). Use it dry or dipped in astringent or hemostatic solution
Photography by Dr. Chu Fountain

## Slide 14
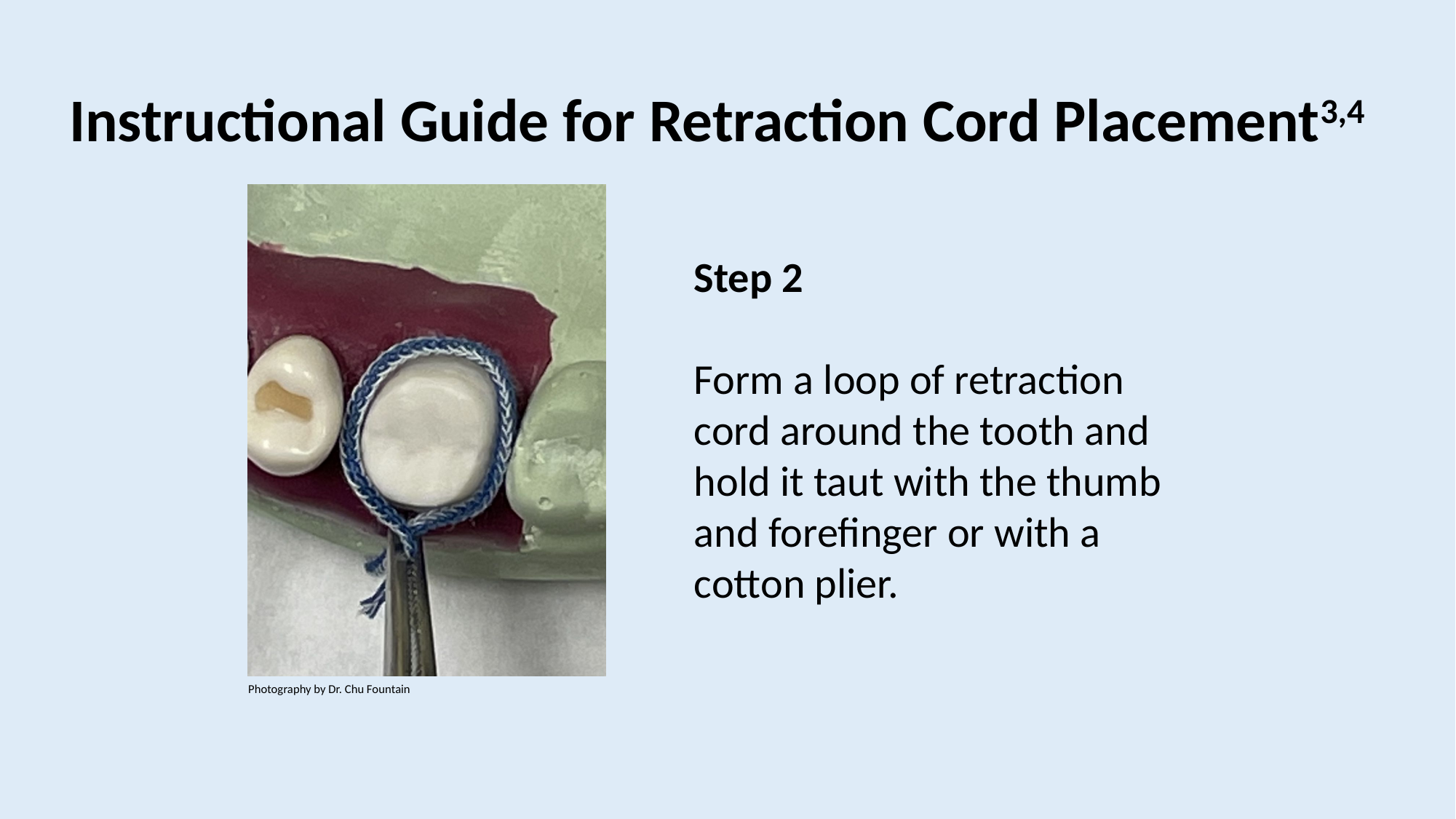

Instructional Guide for Retraction Cord Placement3,4
Step 2
Form a loop of retraction cord around the tooth and hold it taut with the thumb and forefinger or with a cotton plier.
Photography by Dr. Chu Fountain

## Slide 15
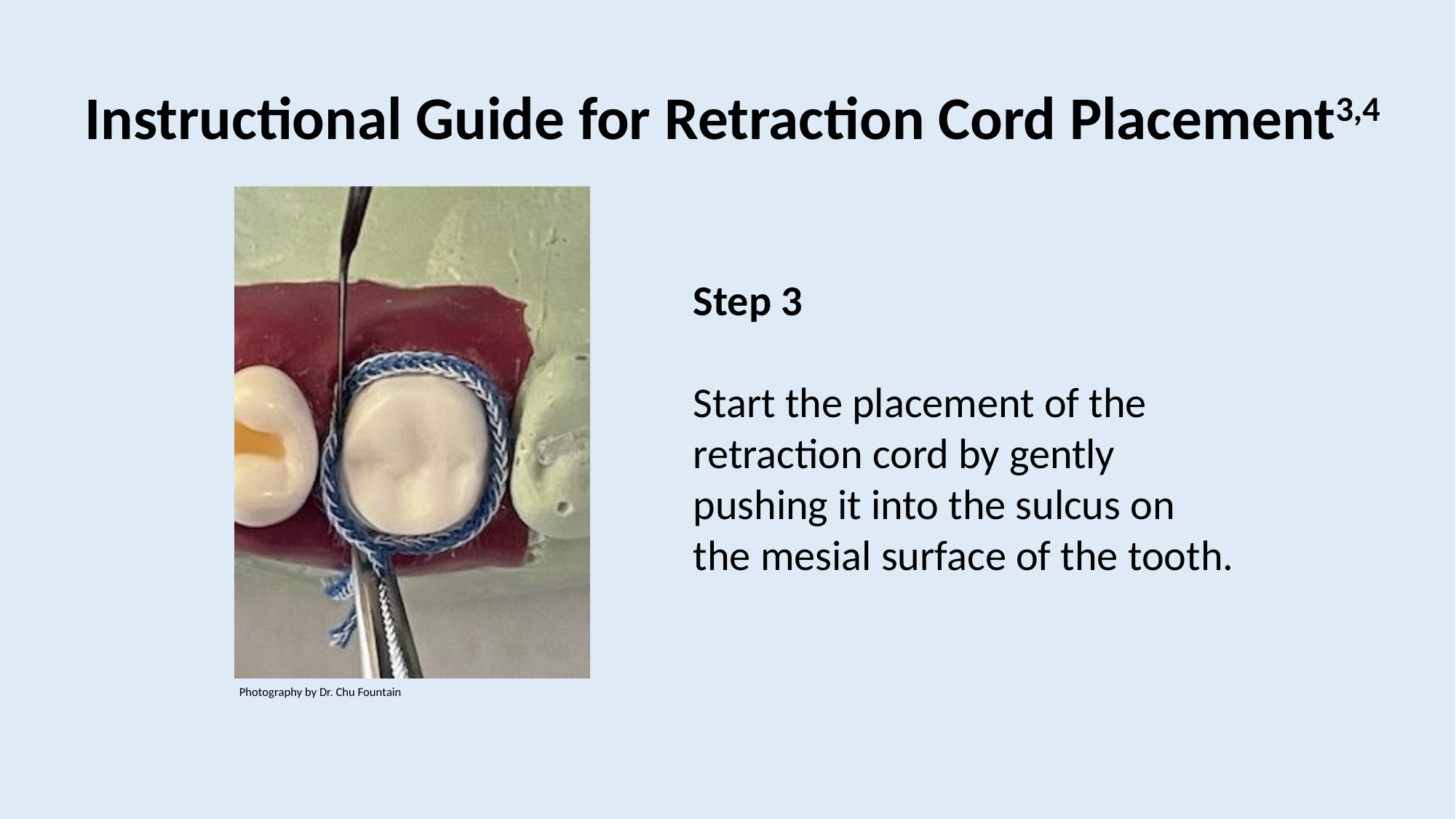

Instructional Guide for Retraction Cord Placement3,4
Step 3
Start the placement of the retraction cord by gently pushing it into the sulcus on the mesial surface of the tooth.
Photography by Dr. Chu Fountain

## Slide 16
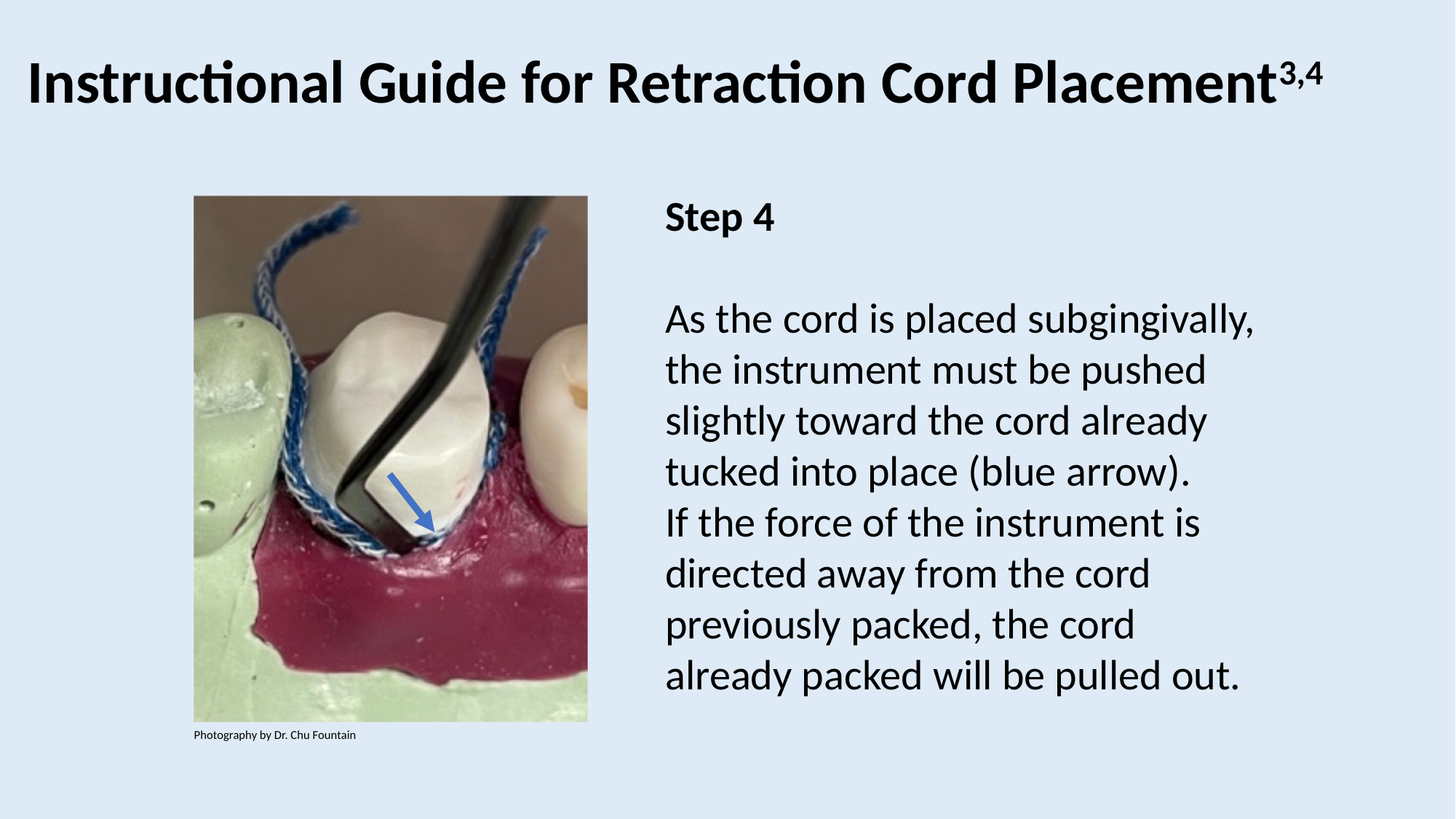

Instructional Guide for Retraction Cord Placement3,4
Step 4
As the cord is placed subgingivally, the instrument must be pushed slightly toward the cord already tucked into place (blue arrow).
If the force of the instrument is directed away from the cord previously packed, the cord already packed will be pulled out.
Photography by Dr. Chu Fountain

## Slide 17
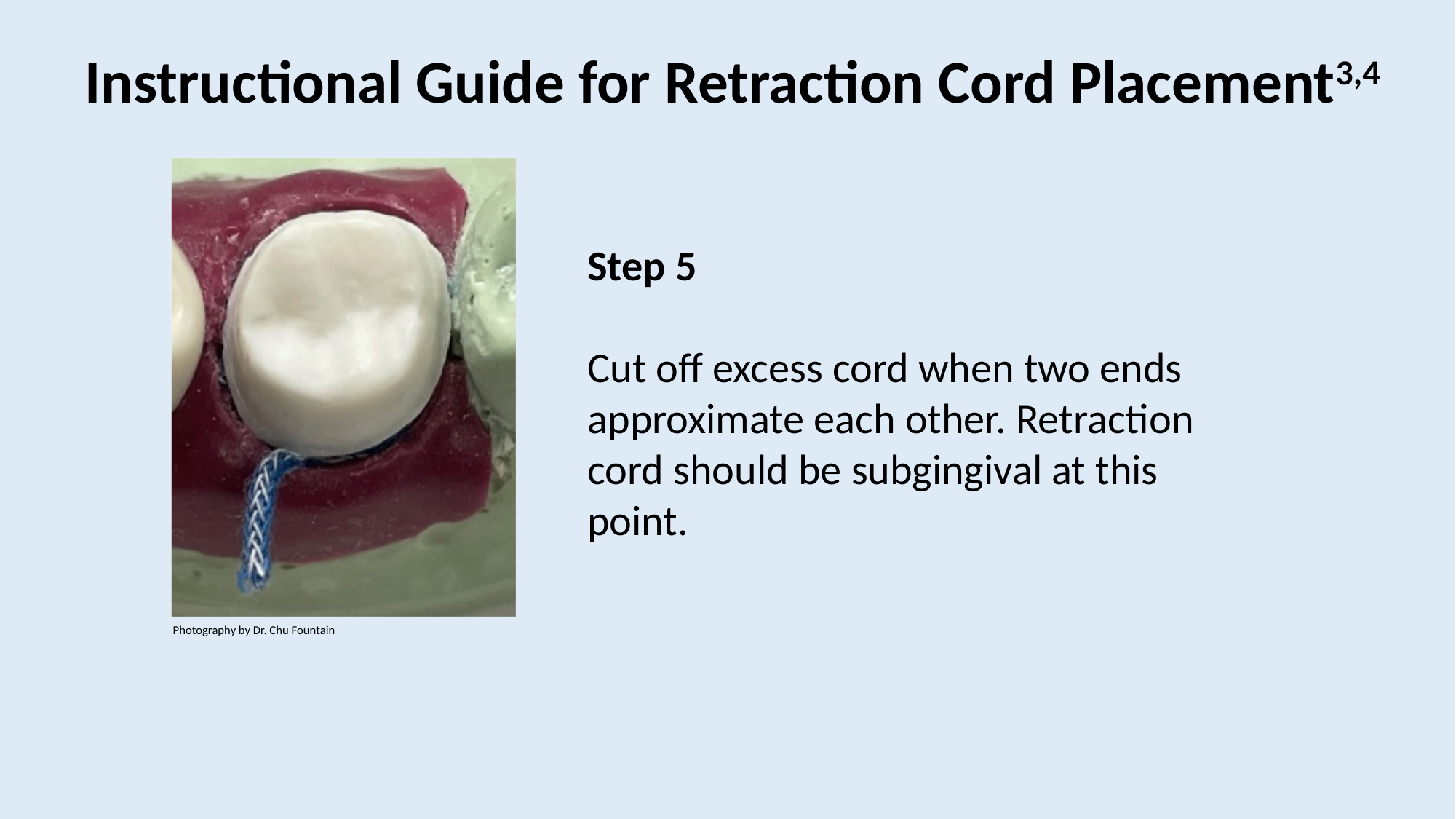

Instructional Guide for Retraction Cord Placement3,4
Step 5
Cut off excess cord when two ends approximate each other. Retraction cord should be subgingival at this point. ​
Photography by Dr. Chu Fountain

## Slide 18
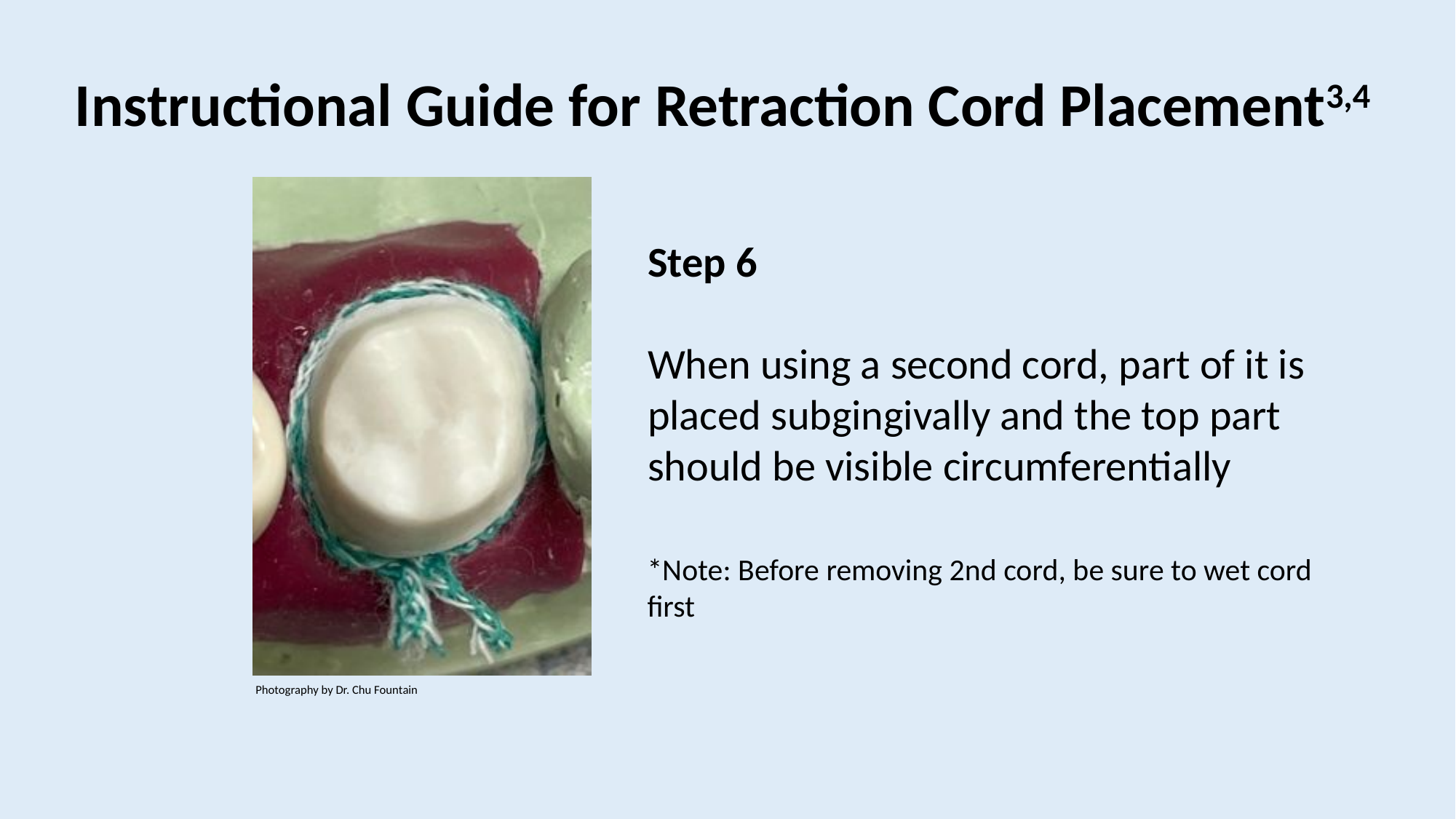

Instructional Guide for Retraction Cord Placement3,4
Step 6
When using a second cord, part of it is placed subgingivally and the top part should be visible circumferentially
*Note: Before removing 2nd cord, be sure to wet cord first
Photography by Dr. Chu Fountain

## Slide 19
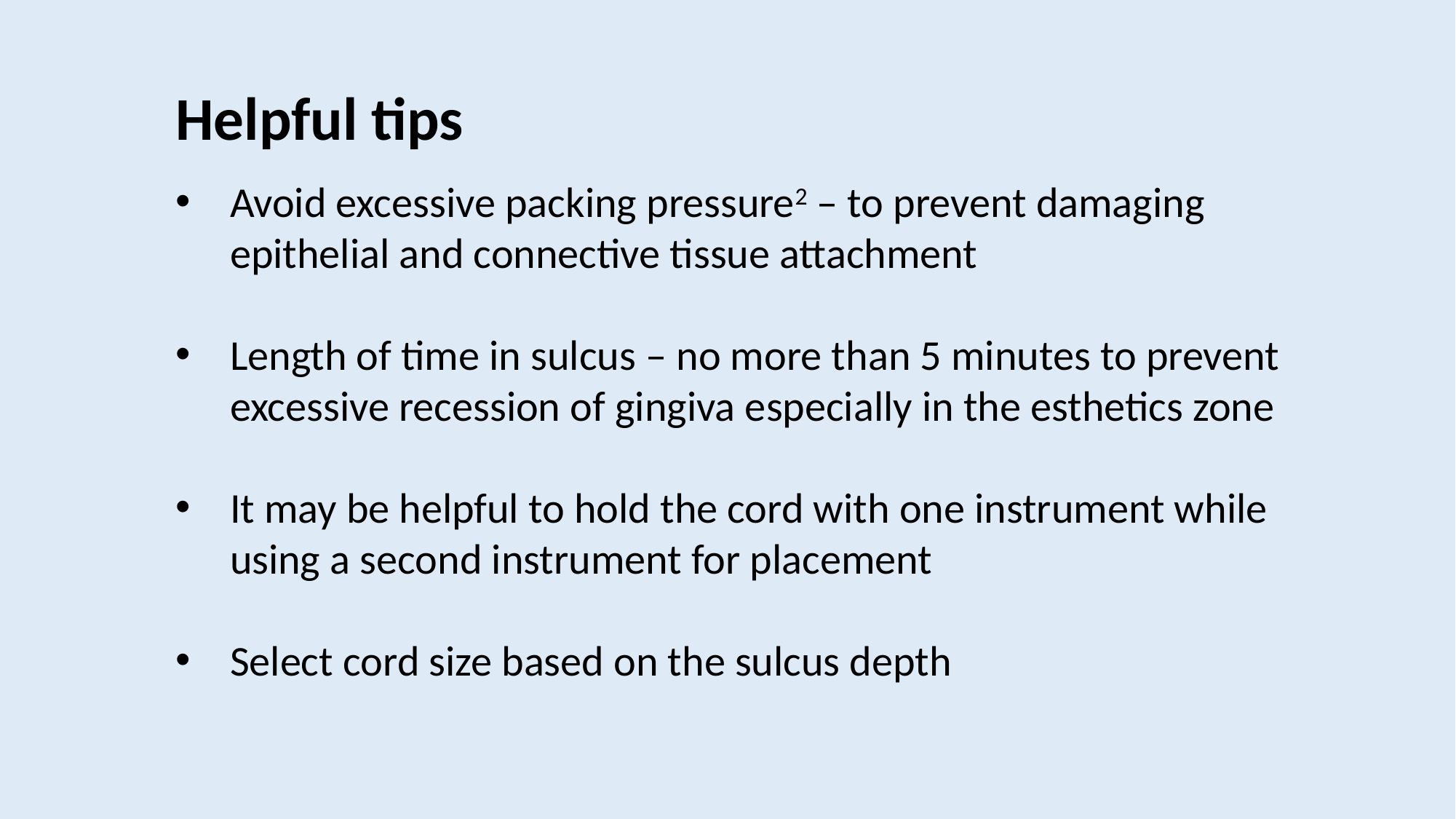

Helpful tips
Avoid excessive packing pressure2 – to prevent damaging epithelial and connective tissue attachment
Length of time in sulcus – no more than 5 minutes to prevent excessive recession of gingiva especially in the esthetics zone
It may be helpful to hold the cord with one instrument while using a second instrument for placement
Select cord size based on the sulcus depth

## Slide 20
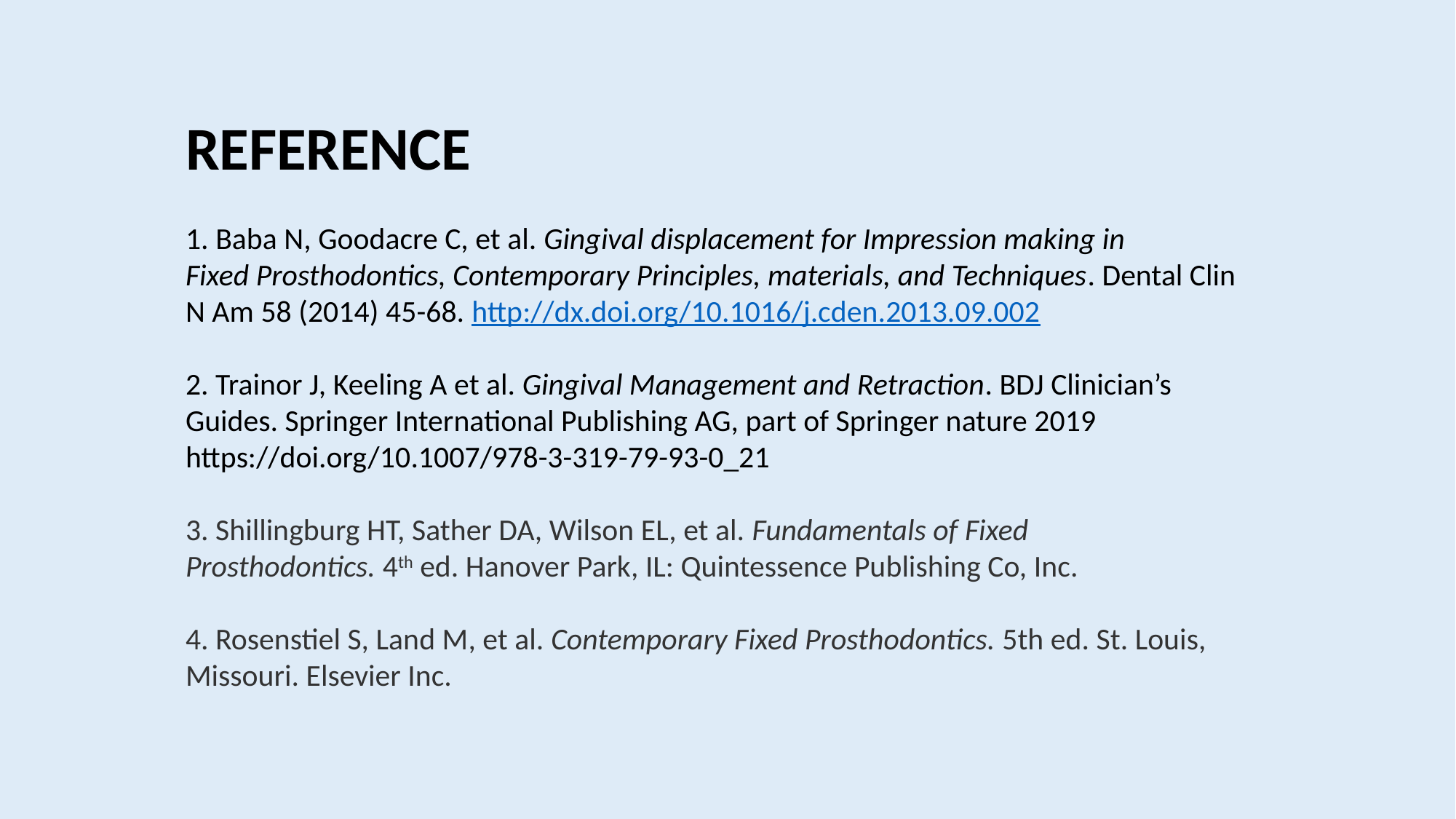

REFERENCE
1. Baba N, Goodacre C, et al. Gingival displacement for Impression making in Fixed Prosthodontics, Contemporary Principles, materials, and Techniques. Dental Clin N Am 58 (2014) 45-68. http://dx.doi.org/10.1016/j.cden.2013.09.002
2. Trainor J, Keeling A et al. Gingival Management and Retraction. BDJ Clinician’s Guides. Springer International Publishing AG, part of Springer nature 2019 https://doi.org/10.1007/978-3-319-79-93-0_21
3. Shillingburg HT, Sather DA, Wilson EL, et al. Fundamentals of Fixed Prosthodontics. 4th ed. Hanover Park, IL: Quintessence Publishing Co, Inc.
4. Rosenstiel S, Land M, et al. Contemporary Fixed Prosthodontics. 5th ed. St. Louis, Missouri. Elsevier Inc.
