# Supplementary material for: Cord Placement Model: An Instructional Guide for Preclinical Dental Students to Practice the Skill of Retraction Cord Placement
Source: MedEdPORTAL. 2023 Feb 28;19:11303. doi: 10.15766/mep_2374-8265.11303 (PMC9971216; doi:10.15766/mep_2374-8265.11303)
Supplement: Supplementary file 1 — Retraction Cord Model Instructional Guide.mp4Instructional Guide for Model Fabrication.docxStudents Instructional Guide.docxFaculty Survey.docxGingival Displacement With Retraction Cord.pptxStudents Instructional Guide Video.mp4Implementation Guide.docxCord Packing Assessment.docxD3 Student Survey.docxD4 Student Survey.docx [file mep_2374-8265.11303-s001.zip › Educational Summary Report_11303.pdf]

MedEdPORTAL® | The AAMC Journal of Teaching and Learning Resources

Original Publication Open Access

Cord Placement Model: An Instructional Guide for Preclinical Dental Students to Practice the Skill of Retraction Cord Placement

Denise Amelia Mills, BSDH, DDS, MHA, EdD*, Anita Chu Fountain, DDS, Daphne Velazquez, DDS

*Corresponding author: dmills@midwestern.edu

Abstract

Introduction: Gingival displacement is essential for ideal margin exposure and improved direct or indirect restorative outcome. Recent literature found many dentists prefer using retraction cord. Some contraindications of other displacement methods make retraction cord displacement preferred. It is necessary to teach dental students appropriate cord placement while minimizing gingival trauma. **Methods:** We developed a stone model using prepared typodont teeth and simulated gingiva that was made of polyvinylsiloxane material. Twenty-three faculty and 143 D2 students were briefed on the instructional guide. After faculty demonstration, D2 students practiced for 10-15 minutes under observation. The following year, former D2 (now D3) and D4 students were asked for feedback on the instructional experience. **Results:** Fifty-six percent of faculty rated the model and instructional guide good to excellent, and 65% rated the student experience as good to excellent, with only one participant rating it poor. Seventy-eight percent of D3 students strongly agreed or agreed the exercise increased their understanding of the technique in placing cord on a patient. Furthermore, 94% of D4 students strongly agreed or agreed having this exercise in preclinical D2 year would have been helpful. **Discussion:** Use of retraction cord to deflect gingiva is still preferred by most dentists. Completing the cord placement exercise on a model helps prepare students to perform the procedure on a patient before arriving at the clinic. Survey comments such as “useful exercise” support use of this instructional model. Overall, faculty and D3 and D4 students felt the exercise was beneficial to use in preclinical education.

Keywords
Cord Packing, Gingival Retraction, Gingival Displacement, Clinical/Procedural Skills Training, Dentistry, Simulation, Dental Education

Educational Objectives

After completion of this educational resource, students will be able to:

1. Describe when the use of cord placement is indicated for gingival displacement.
2. Demonstrate how to manipulate and place gingival retraction cord.
3. Identify appropriate size of retraction cord as well as instruments to use for cord placement.
4. Gain confidence in cord placement for gingival retraction prior to direct patient care.

Citation:
Mills DA, Chu Fountain A, Velazquez D. Cord placement model: an instructional guide for preclinical dental students to practice the skill of retraction cord placement. *MedEdPORTAL*. 2023;19:11303.
https://doi.org/10.15766/mep_2374-8265.11303

Introduction

Gingival displacement (GD), sometimes referred to as retraction, is defined as deflection of the gingival margin away from the tooth.[1] Displacing the gingiva creates adequate vertical and lateral space between the preparation finish line and gingiva to allow the injection of sufficient bulk of impression material into the sulcular space. Also, it can enhance the scanning of gingival margins. Accuracy of an impression and marginal fit of the restoration depend on several aspects, one of those being adequate GD. Gingival retraction is sometimes advantageous when placing direct restorations. Substandard adaptation of restorations with subgingival margins can cause the accumulation of plaque biofilm, leading to gingival inflammation and caries.[2]

Gingival retraction can be achieved by mechanical, chemical, or surgical (electrosurgery or laser) methods. However, laser is contraindicated in the presence of nitrous oxide or where there is a lack of attached gingiva. Retraction cord is the preferred option for GD. It is considered the most common method for gingival management.[3] A 2008 study found that 92% of dentists used

Copyright © 2023 Mills et al. This is an open-access publication distributed under the terms of the Creative Commons Attribution license.

displacement cord, 20% used a soft tissue laser, and 32% used electrosurgery.[4] Another indication for GD with retraction cord is in the cementation of a restoration. The tooth is isolated to help control moisture from gingival crevicular fluid.[5] This also helps to contain the amount of cement pushed into the sulcus. A more recent survey of dentists found the majority continues to use retraction cord for GD due to predictability.[6]

Dental operators who choose cord for retraction must develop safe and effective methods for placement. If the cord is inappropriately manipulated, it can lead to trauma and recession from excessive pressure and the amount of time it is left in the sulcus.[1,4] It is therefore essential for dental programs to teach students the criteria for the gingival retraction technique. Furthermore, when cord is the selected technique, students need to develop the proper approach to cord placement to minimize injury to the gingiva and maintain periodontal health of their patients. Typodonts provided for skills training on simulation manikins traditionally do not have a sulcus for practicing retraction cord placement. Additionally, literature on procedural techniques is lacking, and there are no similar *MedEdPORTAL* publications. Therefore, we identified the need to create a stone model with a sulcus for students to practice retraction cord placement on before providing direct patient care.

Methods

Cord Packing Model Fabrication

We fabricated a stone model with two to six plastic teeth prepared for full crown restorations, as described in the Retraction Cord Model Fabrication Instructional Guide video (Appendix A). Gingival tissue was simulated using medium body polyvinylsiloxane impression material. To maintain cost-effectiveness, teeth that had previously been prepared by students for full crowns were used to fabricate the models. We tested the models with a focus group of five clinical faculty members to preassess them as a teaching tool and aid in developing a postexercise survey. A printed version of the step-by-step Instructional Guide for Fabricating a Retraction Cord Packing Model (Appendix B) was available as an adjunct to the video.

Faculty Perception of Cord Placement Activity

Prior to this simulation activity, we met with the faculty who would be leading the student exercise. Verbal informed consent that described the purpose of their participation in a survey to assess the cord packing activity was given (Institutional Review Board AZ# 1429). We reviewed the students' instructional guide (Appendix C) with faculty as well as the overall goals for the session. To measure the effectiveness of the activity, we distributed a survey (Appendix D) to the faculty to gather their perceptions of the instructional guide and cord packing model as an educational tool.

Implementation

To teach the exercise, we created the cord packing model fabrication video (Appendix A) along with the step-by-step instructional guide (Appendix B). The target audience for this activity was second-year preclinical dental students; the activity itself was an exercise in the simulation laboratory, course number 1636L. The students were just beginning the spring session and preparing for transition to clinic. They had previously received 2 hours of classroom presentations describing measures to facilitate tissue management and fluid control during impression procedures. The video reviewed tissue retraction techniques, when they should be used, and how those results would lead to an acceptable impression.

We created a 20-slide PowerPoint (Appendix E) to use at the commencement of the activity for review. The first portion of the presentation provided background information regarding the use of lasers, cord, and electrosurgery for GD. The second half reviewed cord placement technique. Faculty could determine the appropriate use of the PowerPoint presentation based on student need. We also created a short video of the students' instructional guide (Appendix F) for faculty and/or student reference as needed. The implementation guide (Appendix G) provided overall guidance in facilitating the simulation exercise.

We asked instructors to distribute the instructional guide to the students and verbally review the goals for the exercise. The faculty-to-student ratio was 1:5. Instructors demonstrated the retraction cord placement technique to small groups. Following the demonstration, students received one-on-one opportunities to practice the technique for 10-15 minutes with verbal feedback from faculty. As this session was included as part of a larger daily simulation activity, no formal assessment was provided. The session was a completion exercise supported by verbal faculty feedback. We developed the cord packing assessment (Appendix H) to evaluate students' skill at completion of the exercise.

To gain further understanding of the students' experience, we expanded the activity to a convenience sample of third- and fourth-year students ($n = 71$) the following year. Verbal informed consent that described the purpose of participation was given by the students. The groups surveyed were D3s (formerly D2s) and D4s who had not previously experienced the exercise. The D4s ($n = 34$) were 3 months from completion of their clinical

Copyright © 2023 Mills et al. This is an open-access publication distributed under the terms of the Creative Commons Attribution license.

education. A survey for D3s (Appendix I) and a separate survey for D4s (Appendix J) were used to assess their perception of the exercise. This student cohort had the opportunity to review the instructional guide and practice on the cord placement technique model. The goal of involving the D4 students was to retrospectively determine if they felt it would have been helpful to have had the exercise during their preclinical education.

Results

Faculty Perception of the Cord Packing Exercise
Twenty-three faculty completed the survey following the simulation exercise for cord placement (Table 1). The majority of faculty completing the survey (78%) agreed the model was representative of the experience of placing retraction cord on a patient. Conversely, 21% disagreed or strongly disagreed (Table 1). The overall rating of the model and instructional guide provided a range of responses from poor to excellent. The majority (56%) rated them good to excellent, while 43% rated them fair. The faculty perception of the overall student experience rated more positively, with 65% rating it good to excellent. Thirty percent of faculty gave a fair rating, and only one survey participant rated the experience as poor (Table 1).

Students' Perception of the Cord Packing Exercise
The exercise was first presented to a D2 class of 143 students. There was no formal assessment, so the actual number of participants could not be identified. Some of the students who had completed the cord placement exercise in the D2 year were surveyed in their third year ($n = 37$) of clinic (Table 2). We surveyed these students 9 months after their full-time transition into clinic. Forty-nine percent of these participants strongly agreed or agreed that the training model in the D2 year prepared them for placing cord on patients, while 17% had no opinion. Twenty-six percent of the students disagreed (Table 2). Of the D4 students ($n = 34$, Table 3), 94% strongly agreed or agreed that having the exercise in preclinical D2 year would have been helpful. One of the 34 participants had no opinion of the exercise, while one participant disagreed that it was helpful (Table 3).

We read the open-ended survey comments of D4 students and faculty to identify recurring themes (Table 4). Most comments were related to the retraction cord packing model design. The second most prominent theme centered around the model activity as a learning resource. Comments regarding the instrument used for the exercise came only from the faculty. There were several comments from students regarding performing cord placement in vivo.

Discussion

Gingival retraction cord is used to provide the dentist with a clear working view of a tooth and its prepared margins prior to taking the impression. This can be captured with impression material or scanned digitally. In one study, although in vitro, Son and Lee[7] discovered a 63% improved accuracy of intraoral scans of subgingival margins with the use of GD cord. Becoming competent in retraction cord placement is an essential skill for enhancing restoration success even in the case of digital dentistry. Gottlieb, Vervoorn, and Buchanan[8] suggested that an acceptable level of competence should be obtained prior to clinical patient care because most dental procedures are irreversible and learning them solely on patients is not a suitable practice. In our survey, 74% of D4 student participants strongly agreed or agreed that the model was representative of the experience of placing retraction cord on a patient. However, approximately one-fourth (26%) of D3 students disagreed that the model prepared them for placing cord on a patient. There could be several reasons for this response. The activity that day was one of several exercises of the daily session and was not assessed upon completion; hence, there was no record of which students completed the exercise. Some students mentioned that they were not present that day and therefore did not have the chance to practice in their D2 year simulation lab.

Traditionally, our students learn how to pack retraction cord from on-the-job training when they arrive at the clinic. While our exercise activity is not an exact replication of cord placement on a patient, it allows students to gain knowledge of a set of skills that

Table 1. Faculty Responses ($n = 23$)

Item Surveyed	Strongly Agree	Agree	No Opinion	Disagree	Strongly Disagree	Excellent	Good	Fair	Poor
Retraction cord placement model									
Model is representative of placing retraction cord on a patient.	0%	78%	0%	17%	4%				
The students' instructional guide is a beneficial adjunct to the model.	17%	52%	26%	4%	0%				
Student experience of using the model									
Overall rating of the model and instructional guide.						13%	43%	43%	0%
Overall impression of students' experience of using the instructional model and guide.						26%	39%	30%	4%

Copyright © 2023 Mills et al. This is an open-access publication distributed under the terms of the Creative Commons Attribution license.

Table 2. Third-Year Dental Student Responses ($n = 37$)

Item Surveyed	Strongly Agree	Agree	No Opinion	Disagree	Strongly Disagree	Excellent	Good	Fair	Poor
Retraction cord placement model									
Training model in D2 year prepared me for placing on patient in clinic.	20%	29%	17%	26%	9%				
The model and instructional guide were useful learning tools.	33%	56%	6%	6%	0%				
The exercise increased my understanding of appropriate technique for cord placement.	31%	47%	17%	6%	0%				
The exercise enhanced my confidence in clinic.	24%	30%	30%	16%	0%				
Instructional guide and cord packing model									
Overall rating of the model and instructional guide.						38%	43%	19%	0%
Overall rating of cord placement exercise experience.						35%	38%	27%	0%

Abbreviation: D2, second-year.

they would otherwise learn only on their first patient encounters. This set of skills includes the proper instruments to use, as well as appropriate technique to pack the cord with minimal trauma to the gingiva. Comments from faculty such as “not realistic but good exercise” and “doesn’t simulate same feeling of placing cord but still useful exercise” support the use of this instructional model. Furthermore, dental student comments such as “good but doesn’t mimic gingiva, good starter,” and “closest representation of what it is like to place cord” are further support of the need for using the cord placement model for training.

Dental students were also given the opportunity to experience using more than one type of cord placement instrument. One repeated comment from faculty survey participants related to the serrated cord placement instrument used for the exercise. It was described as “poor” and was said to have easily “pulled [the] cord out”; one commenter “rec[ommended] non serrated cord packer.” Interestingly, the D3 and D4 students commented that they liked using the serrated cord placement instrument better than the plain Teflon-coated one available in the clinic. Because of their comments, we are considering offering more than one type of cord placement instrument in the future.

Based on participant responses regarding the instructional model, we modified the fabrication technique before presenting the exercise to the third- and fourth-year students. Due to faculty comments such as “[did] gingivectomy to make sulcus, then worked well,” and “need sulcus, difficult to pack cord,” we created a sulcus on the model before presenting to the D3 and D4 students. Those modifications are reflected in the model fabrication video and step-by-step guide (Appendices A and B). Faculty members from the original focus group that assessed the model evaluated the revised model and felt the revision of the simulated sulcus was a better representation of the clinical experience.

A limitation of the evaluation approach in our exercise was the use of two different iterations of the model design. Faculty feedback gathered was based on the initial model design without the sulcus. The same models were used with the D2 students who participated in the first activity. After we modified the model design with a sulcus, feedback from the D3 (former D2) and D4 students was positive, although several students commented that one big challenge of cord placement was having to use indirect vision. The inability to mount the model into a manikin is another limitation to the cord packing exercise. We will consider taking on the challenge of developing a technique for mounting the model.

Broader implications for including this activity in preclinical simulation are the skill and practice required to place retraction cord. Improper cord placement can cause gingival injury and damage to the sulcular epithelium. This can lead to recession

Table 3. Fourth-Year Dental Student Responses ($n = 34$)

Item Surveyed	Strongly Agree	Agree	No Opinion	Disagree	Strongly Disagree	Excellent	Good	Fair	Poor
Retraction cord placement model									
The model is representative of the experience of placing retraction cord on a patient.	12%	62%	15%	12%	0%				
It would have been helpful to have this exercise in preclinical D2 year.	59%	35%	3%	3%	0%				
Instructional guide and cord packing model									
Overall rating of the model and instructional guide.						27%	55%	18%	0%
Overall rating of experience of placing retraction cord.						24%	55%	18%	3%

Abbreviation: D2, second-year.

Copyright © 2023 Mills et al. This is an open-access publication distributed under the terms of the Creative Commons Attribution license.

Table 4. Thematic Content Analysis of Fourth-Year Dental Student and Faculty Open-Ended Survey Responses

Theme	Student Quotes	Faculty Quotes
Factors associated with cord packing model design	"Needs sulcus."<br>"Better experience with sulcus."<br>"Very useful, feels like a real sulcus."<br>"Model needs to have sulcus."<br>"Pvs [polyvinylsiloxane] is not flexible to represent gingiva."<br>"Better than nothing, need more vertical pressure."<br>"Model needs to have sulcus."<br>"Requires more force to place on the model."	"Gingivectomy, cord popping up, frustration."<br>"Need sulcus, difficult to pack cord."<br>"Gingivectomy to make sulcus, then worked well."<br>"Use mylar strip to create sulcus."<br>"Allowed cord to be packed easier at the end of the day."<br>"Suggested different material for gingiva."<br>"Difficult to replicate gingiva."<br>"Vps [polyvinylsiloxane] tore interproximally, not well adapted on some preps."
Instruments used for the exercise		"Rec[ommend] non serrated cord packer."<br>"Cord packer instrument is poor."<br>"Cord packer pulled cord out."
Consideration of cord placement exercise as a student learning resource	"Helpful for D2."<br>"Very helpful."<br>"Closest representation of what it's like to place cord."<br>"Good but doesn't mimic gingiva, good starter."	"Good introduction to cord packing, not realistic but good exercise."<br>"Doesn't simulate same feeling of placing cord but still useful exercise."<br>"Great exercise."
Suggestions for direct patient experience	"Try on each other instead of a model."<br>"No cheeks, tongue, bleeding etc."	"Practice on real people."
Additional comment	"More helpful if they had a lecture in D2."	

Abbreviations: D2, second-year.

and unwanted exposure at the margin of a restoration. Based on the faculty and student comments and a lack of teaching resources for cord placement, we believe this exercise should be incorporated into the dental simulation curriculum.

Appendices

A. Retraction Cord Model Instructional Guide.mp4

B. Instructional Guide for Model Fabrication.docx

C. Students Instructional Guide.docx

D. Faculty Survey.docx

E. Gingival Displacement With Retraction Cord.pptx

F. Students Instructional Guide Video.mp4

G. Implementation Guide.docx

H. Cord Packing Assessment.docx

I. D3 Student Survey.docx

J. D4 Student Survey.docx

All appendices are peer reviewed as integral parts of the Original Publication.

Denise Amelia Mills, BSDH, DDS, MHA, EdD: Associate Professor and Clinical Care Coordinator, Midwestern University College of Dental Medicine-Arizona; ORCID: https://orcid.org/0000-0002-7784-3941

Anita Chu Fountain, DDS: Assistant Professor and Clinical Care Faculty, Midwestern University College of Dental Medicine-Arizona

Daphne Velazquez, DDS: Assistant Professor and Preclinical Faculty, Midwestern University College of Dental Medicine-Arizona

Acknowledgments
Thanks to Dean Brad Smith and the Midwestern University College of Dental Medicine-Arizona for supporting this project.

Disclosures
None to report.

Funding/Support
None to report.

Ethical Approval
The Midwestern University Office of Research Institutional Review Board deemed further review of this project not necessary.

References

1. Trainor J, Keeling A, Wassell R. Gingival management and retraction. In: Wassell R, Nohl F, Steele J, Walls A, eds. *Extra-Coronal Restorations: Concepts and Clinical Application*. 2nd ed. Springer; 2019:363-374.
2. Mjör IA, Moorhead JE, Dahl JE. Reasons for replacement of restorations in permanent teeth in general dental practice. *Int Dent J*. 2000;50(6):361-366. https://doi.org/10.1111/j.1875-595X.2000.tb00569.x
3. Azzi R, Tsao TF, Carranza FA Jr, Kenney EB. Comparative study of gingival retraction methods. *J Prosthet Dent*. 1983;50(4): 561-565. https://doi.org/10.1016/0022-3913(83)90581-4

Copyright © 2023 Mills et al. This is an open-access publication distributed under the terms of the Creative Commons Attribution license.

4. Ahmed SN, Donovan TE. Gingival displacement: survey results of dentists' practice procedures. *J Prosthet Dent*. 2015;114(1): 81-85.E2. https://doi.org/10.1016/j.prosdent.2014.11.015
5. Santos GC Jr, Santos MJMC, Rizkalla AS. Adhesive cementation of etchable ceramic esthetic restorations. *J Can Dent Assoc*. 2009;75(5):379-384.
6. Al Hamad KQ, Azar WZ, Alwaeli HA, Said KN. A clinical study on the effects of cordless and conventional retraction techniques on the gingival and periodontal health. *J Clin Periodontol*. 2008; 35(12):1053-1058. https://doi.org/10.1111/j.1600-051X.2008.01335.x
7. Son K, Lee KB. Effects of finish line locations of tooth preparation on the accuracy on intraoral scanners. *Int J Comput Dent*. 2021;24(1):29-40.
8. Gottlieb R, Vervoorn JM, Buchanan J. Simulation in dentistry and oral health. In: Levine AI, DeMaria S, Schwartz AD, Sim AJ, eds. *The Comprehensive Textbook of Healthcare Simulation*. Springer; 2013:329-340. https://doi.org/10.1007/978-1-4614-5993-4_21

Received: March 11, 2022
Accepted: November 21, 2022
Published: February 28, 2023

Copyright © 2023 Mills et al. This is an open-access publication distributed under the terms of the Creative Commons Attribution license.
