# Supplementary material for: Cord Placement Model: An Instructional Guide for Preclinical Dental Students to Practice the Skill of Retraction Cord Placement
Source: MedEdPORTAL. 2023 Feb 28;19:11303. doi: 10.15766/mep_2374-8265.11303 (PMC9971216; doi:10.15766/mep_2374-8265.11303)
Supplement: Supplementary file 1 — Retraction Cord Model Instructional Guide.mp4Instructional Guide for Model Fabrication.docxStudents Instructional Guide.docxFaculty Survey.docxGingival Displacement With Retraction Cord.pptxStudents Instructional Guide Video.mp4Implementation Guide.docxCord Packing Assessment.docxD3 Student Survey.docxD4 Student Survey.docx [file mep_2374-8265.11303-s001.zip › G. Implementation Guide.docx]

**Implementation Guide**

**Practical implementation**

Materials needed

Instructional power point

Instructional video

Cord packing exercise cast

Serrated and/or Teflon coated or plain flat sided instrument with rounded tip

Scissors

Cotton pliers

Cord Packing Assessment sheet (Appendix H)

Pencil or pen

Flat surface with adequate lighting and free of other materials for students to work on

**Length of the session**

- Power point presentation: 10 minutes
- Video presentation: 5 minutes
- Cord packing exercise: 15 minutes per student pair

**Faculty/facilitator needs/student needs:**

- Refer to materials needed section above
- Additional to that have a computer or tablet that can display the power point and video

**Preparation needs:**

- Review power point and video prior to student exercise. Ensure all media works properly by testing power point and video display.
- Verify that all the materials are available and ready. Ideally there should be a cast for every 2 students.

**Cord packing exercise steps: (use Cord Packing Assessment as an additional reference)**

1. Cut off a 2-inch piece of the desired cord. Students should determine the appropriate size based on the location of the margin (subgingival versus equigingival), type of cord packing technique to be used (1 versus 2-cord technique) and sulcus size.
2. Form a loop of retraction cord around the tooth and hold it taut.
3. Begin placement of the cord starting on the mesial surface and continuing circumferentially directing the forces slightly toward the cord that is already in place. Care should be taken not to exert too much pressure. Students can try both serrated and non-serrated instruments.
4. Cut off the excess cord when the two ends approximate each other but do not overlap or leave a gap in between them.
5. If doing the 2-cord technique repeat the process with the larger cord.
6. To remove the cord: use cotton pliers to remove the cord slowly. Students should be reminded that on a patient the cord should be moistened prior to removal.

**Tips for successful deployment:**

- Discuss clinical tips and explanations during each step.
- It might be helpful for some students to hold the cord with one instrument while using a second instrument for placement.
- Collect all materials and store together for the next use.

**Limitations for resources:**

- If there are not enough models available for every 2 students, they can be shared among several students. Caution should be taken not to damage the gingiva-like material with overuse or excessive pressure.

**Ideas for expanding/improving this educational resource:**

- The cast could be adapted to be placed inside a DEXTER to provide the student with practice inside the simulated patient’s mouth.
