# Supplementary material for: Cord Placement Model: An Instructional Guide for Preclinical Dental Students to Practice the Skill of Retraction Cord Placement
Source: MedEdPORTAL. 2023 Feb 28;19:11303. doi: 10.15766/mep_2374-8265.11303 (PMC9971216; doi:10.15766/mep_2374-8265.11303)
Supplement: Supplementary file 1 — Retraction Cord Model Instructional Guide.mp4Instructional Guide for Model Fabrication.docxStudents Instructional Guide.docxFaculty Survey.docxGingival Displacement With Retraction Cord.pptxStudents Instructional Guide Video.mp4Implementation Guide.docxCord Packing Assessment.docxD3 Student Survey.docxD4 Student Survey.docx [file mep_2374-8265.11303-s001.zip › H. Cord Packing Assessment.docx]

**Cord Packing Assessment**

**Assessment Criteria**

**3** – Student meets most of the criteria without assistance.

**2** – Student requires assistance to meet the stated criteria.

| **Criteria** | **Self** | **Instructor** |
| --- | --- | --- |
| **Placing the cord** |  |  |
| 1. Student selected appropriate instrument to use for cord placement. |  |  |
| 1. Student measured “sulcus” with a probe and selected appropriate cord size dependent on sulcus. |  |  |
| 1. Student demonstrated proper instrument angulation (tucks cord toward cord already tucked in place) |  |  |
| 1. Once the cord is placed the two ends are approximated (no more than one millimeter deficiency or excess) |  |  |
| 1. If adequate “sulcus” depth for two-cord technique, student selects appropriate cord sizes. If second cord is required, it is placed above first cord and can be seen circumferentially. |  |  |
| **Removing the cord** |  |  |
| 1. Grasp the end of the retraction cord with cotton plier and remove |  |  |

**1** – Student did not prepare accordingly for the stated criteria.
