# Supplementary material for: Cord Placement Model: An Instructional Guide for Preclinical Dental Students to Practice the Skill of Retraction Cord Placement
Source: MedEdPORTAL. 2023 Feb 28;19:11303. doi: 10.15766/mep_2374-8265.11303 (PMC9971216; doi:10.15766/mep_2374-8265.11303)
Supplement: Supplementary file 1 — Retraction Cord Model Instructional Guide.mp4Instructional Guide for Model Fabrication.docxStudents Instructional Guide.docxFaculty Survey.docxGingival Displacement With Retraction Cord.pptxStudents Instructional Guide Video.mp4Implementation Guide.docxCord Packing Assessment.docxD3 Student Survey.docxD4 Student Survey.docx [file mep_2374-8265.11303-s001.zip › I. D3 Student Survey.docx]

**D3 Student Survey**

**D3 student’s perception in the assessment of the instructional training guide for retraction cord placement**

Please respond to the following questions based on the Likert scale of 1-5

1 – Strongly agree

2 – Agree

3 – No opinion

4 – Disagree

5 – Strongly disagree

1. The training exercise in placing retraction cord in model during simulation clinic in D2 year prepared me for placement on patients in the clinic setting.

2. The model and instructional guide were useful learning tools.

3. The exercise increased my understanding of appropriate technique in retraction cord placement.

4. The exercise enhanced my confidence in cord placement on a patient in clinic.

Please use the 1 to 4 rating for the next two questions.

1 – Excellent

2 – Good

3 – Fair

4 – Poor

5. What is your overall rating of the model and the instructional guide?

6. What is your overall rating of the cord placement exercise experience?
