# Supplementary material for: Cord Placement Model: An Instructional Guide for Preclinical Dental Students to Practice the Skill of Retraction Cord Placement
Source: MedEdPORTAL. 2023 Feb 28;19:11303. doi: 10.15766/mep_2374-8265.11303 (PMC9971216; doi:10.15766/mep_2374-8265.11303)
Supplement: Supplementary file 1 — Retraction Cord Model Instructional Guide.mp4Instructional Guide for Model Fabrication.docxStudents Instructional Guide.docxFaculty Survey.docxGingival Displacement With Retraction Cord.pptxStudents Instructional Guide Video.mp4Implementation Guide.docxCord Packing Assessment.docxD3 Student Survey.docxD4 Student Survey.docx [file mep_2374-8265.11303-s001.zip › J. D4 Student Survey.docx]

**D4 Student Survey**

**D4 student perception in the assessment of the retraction cord model as an instructional tool survey**

Please respond to the following questions based on the Likert scale of 1-5

1 – strongly agree

2 – agree

3 – no opinion

4 – disagree

5 – strongly disagree

1. The model is representative of the experience of placing retraction cord on a patient.

2. It would have been helpful to have this exercise in pre-clinical D2 year before entering the clinic

Please use the 1 to 4 rating scale for the next two questions.

1 – Excellent

2 – Good

3 – Fair

4 – Poor

3. What is your overall rating of the model and instructional guide?

4. As a student, how would you rate the overall experience of placing retraction cord on models?

5. Please share additional comments__________________________________________
